# Beyond central‐tendency: If we agree discrete vegetation communities do not exist, should we investigate other methods of clustering?

**DOI:** 10.1002/ece3.10757

**Published:** 2023-11-20

**Authors:** Mark G. Tozer, David A. Keith

**Affiliations:** ^1^ NSW Department of Planning and Environment Parramatta New South Wales Australia; ^2^ School of Biological, Earth and Environmental Science, Centre for Ecosystem Science University of NSW Sydney New South Wales Australia

**Keywords:** classification stability, clustering, CLUTO, community theory, essentialism, graph theory, network theory, reification, vegetation classification, vegetation databases

## Abstract

Clustering is indispensable in the quest for robust vegetation classification schemes that aim to partition, summarise and communicate patterns. However, clustering solutions are sensitive to methods and data and are therefore unstable, a feature that is usually attributed to noise. Viewed through a central‐tendency lens, noise is defined as the degree of departure from type, which is problematic since vegetation types are abstractions of continua, and so noise can only be quantified relative to the particular solution at hand. Graph theory models the structure of vegetation data based on the interconnectivity of samples. Through a graph‐theoretic lens, the causes of instability can be quantified in absolute terms via the degree of connectivity among objects. We simulated incremental increases in sampling intensity in a dataset over five iterations and assessed classification stability across successive solutions derived using algorithms implementing, respectively, models of central‐tendency and interconnectivity. We used logistic regression to model the likelihood of a sample changing groups between iterations as a function of distance to the centroid and degree of interconnectivity. Our results show that the degree to which samples are interconnected is a more powerful predictor of instability than the degree to which they deviate from their nearest centroid. The removal of weakly interconnected samples resulted in more stable classifications, although solutions with many clusters were apparently inherently less stable than those with few clusters, and improvements in stability flowing from the removal of outliers declined as the number of clusters increased. Our results reinforce the fact that clusters abstracted from continuous data are inherently unstable and that the quest for stable, fine‐scale classifications from large regional datasets is illusory. Nevertheless, our results show that using models better suited to the analysis of continuous data may yield more stable classifications of the available data.

## Graphical abstract

Clustering is indispensable to the task of building robust vegetation classification systems, and yet the attribution of samples to clusters is notoriously unstable. The likelihood of a sample relocating to a new cluster (instability) upon the addition of new data is inversely proportional to the degree to which it is interconnected with other samples (A&C). Conversely, proximity to the cluster centroid is a poor predictor of instability because cluster centroids are mobile (B&D).

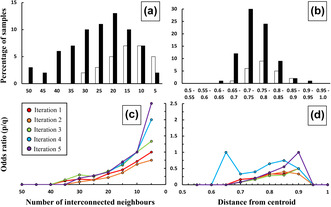

## 1 INTRODUCTION

Vegetation classification systems have become a critical resource for ecological research and management (van der Maarel & Franklin, 2013). They support a wide range of conservation applications such as habitat classification (Chytrý & Tichý, 2018), ecosystem risk assessment (Rodríguez et al., 2015), development of government policy and environmental regulation (Botts et al., 2020), calculation of ecosystem offsets (Sonter et al., 2017), assessment of habitat loss (Skowno et al., 2021) and setting restoration targets (Macfarlane et al., 2017). Robust classifications (those that remain relatively stable with the addition of new data) are critical to the integrity of these applications. Yet clustering, one of the most important tools for interpreting patterns in vegetation data objectively, produces solutions that are highly sensitive to changes in data and analysis procedures and are hence unstable (Tichy et al., 2014). Instability is manifested in one‐to‐many relationships between the clusters emerging from alternative treatments of the data in any given domain (Peet & Roberts, 2013) and is usually attributed to ‘noise’.

Noise, in the general sense, is disorder or randomness in data caused by factors that degrade signals. In the context of central‐tendency models, noise is quantified as the degree of deviation from type (Simberloff, 1980), where type may represent a centroid or any other reference point within a group of objects. Whittaker (1978) explained the particular noisiness of vegetation types by invoking comparisons with the taxonomy of individual organisms. Individuals of a particular species, he argued, possess many characteristics in common and are clearly differentiated from other species, with few individuals exhibiting characteristics intermediate between species. In comparison, he postulated that intergradation between vegetation types is common, and so samples vary to different degrees from putative types. While the degree to which taxonomic and syntaxonomic data differ in this respect is open to debate (Moravec, 1989), vegetation data are notable for containing non‐trivial subsets of samples with poor resemblance to recognised types (Wiser & De Cáceres, 2013). Even so, humans remain deeply connected to and dependent on class‐concepts for interpreting and communication patterns in nature (Keith et al., 2022). Therefore, whatever the flaws in this model, exploring ways to make vegetation types abstracted from continuous data as robust as possible, remains an essential endeavour for communicating about nature.

The concept of noise can potentially be leveraged to develop more stable vegetation classifications by differentiating between inherent variation among the members of a cluster and deviations from type that are so extreme that the efficacy of any attribution to type is questionable (outliers). Noise clustering (De Cáceres et al., 2010; Wiser & De Cáceres, 2013) is conceptually the most flexible approach to the problem because it allows for samples to be categorised as either intermediate between clusters (transitional samples) or as noise (outliers, remote from all clusters). This captures the essence of noise as deviation from type (Simberloff, 1980) as well as the concept of outliers, for example samples affected by historical events that render them dissimilar to all other samples.

While the concepts of transitional and outlier samples appear simple and intuitive, they are problematic in the context of vegetation science. The problem arises because the concept of deviation from type explicitly requires acknowledging the existence of vegetation types (Wiegleb, 1989), and yet most researchers and naturalists accept the continuum theory of vegetation as central to ecology (Austin, 1985, 1986). Continuum theory is founded on reductionist principles enunciated independently by both Ramenskij (1924) and Gleason (1926). It holds that species are distributed independently on resource and climatic gradients (Austin & Smith, 1989), and recognisable, repeated combinations of species occur due to the coincidence of their resource requirements and physiological tolerances (Moravec, 1989). Continuum theory was erected in opposition to discrete models of vegetation, which hold that vegetation is discontinuous and manifests as a finite set of discrete communities. While a discrete model of communities is most strongly associated with Clements's (1916) contentious climax concept (Eliot, 2007), it is central to traditional phytosociology (Austin, 2013) and is recognisable in Tansley's quasi‐organism (Tansley, 1920) and ecosystem (Tansley, 1935) concepts as well as the integrated concept (Moravec, 1989).

While complete independence among species is unlikely due to competition and other interactions (Keddy & Laughlin, 2021), there is ample evidence that the distributions of species occupy broadly overlapping ranges along gradients with asynchronous optima (Whittaker, 1975). Conversely, perceived discontinuities can be explained by the expression of environmental gradients in real geographical space (Austin & Smith, 1989) and the clustering of species' boundaries on steep gradients (Shipley & Keddy, 1987; van der Maarel & Franklin, 2013). From the reductionist perspective, therefore, classification is the process of identifying abstract entities that delimit parts of the continuum (De Cáceres & Wiser, 2012). Each classification reflects the quantity and geographic distribution of the available data (Mucina, 1997), and so noise, in this context, is characterised as deviation from patterns in the structure of the data (centroids) as opposed to deviation from type (Wiegleb, 1989).

While the distinction may appear semantic, it highlights a fundamental bipolarity between discrete and continuum models of vegetation. Under the continuum model, there is no expectation of stability in classification (Mucina, 1997). It is widely acknowledged that the same data can yield multiple, equally informative classification solutions under different analysis treatments (Moravec, 1989; Mucina, 1997; Tichy et al., 2014; Wiser & De Cáceres, 2013), and so the aim is simply to seek stable classifications of the available data (Feoli & Lausi, 1981).

Through a central‐tendency lens, whether or not a sample constitutes noise is thus entirely a function of the particular solution at hand. That is, a sample may theoretically present as noise under one parametrisation of analysis and not under another. A sample can only be categorised as noisy in an absolute sense with reference to type concepts that are implicitly assumed to be stable but are not. Central‐tendency methods suffer from this problem by requiring noise to be quantified in relation to a particular clustering solution. Noise clustering, furthermore, reinforces the expectation that, contrary to continuum theory, stable clusters exist, between which transitional samples may lie. A particular limitation of this model concerns the fact that the operator essentially decides the degree of noisiness inherent in the data, choosing the value of the noise parameter to ensure not too many samples are omitted (Wiser & De Cáceres, 2013).

We argue this bipolarity in relation to theory—accepting continuum theory but modelling various properties of vegetation as though they are discontinuous—hinders the development of stable classifications because noisiness cannot be quantified in any objective or absolute sense to provide guidance in the development of classification systems. If outliers contribute to the instability of classification schemes, then the removal of samples that are noisy in an absolute sense should aid in the retrieval of stable patterns in the data.

We propose graph theory (also known as network theory) as an alternative approach that avoids some of the limitations of central‐tendency approaches. Graphs are mathematical structures that model pairwise relationships between samples (referred to from here on as interconnectivity) as opposed to relationships between group members and a theoretical average state (central‐tendency). In the context of vegetation science, a graphical model comprises a set of vertices, one for each plot sample (releve) contained in the dataset. Edges (junctions) between pairs of vertices represent relationships between samples and can be either binary (e.g. pairwise similarity exceeds a specified threshold) or weighted (e.g. degree of similarity, number of neighbours in common). Thresholds or weights may be used as criteria to create a sparse graph, so‐called because it contains many fewer edges than the maximum possible (Han et al., 2012). Patterns in the data (i.e. clusters) are represented as interconnected networks, identified by variation in the density of edges (or their weights) among vertices (samples) in the graph. Conceptually, the approach is aligned with boundary‐based approaches, which are commonly implemented using hierarchical or agglomerative algorithms with merge/split decisions determined on the basis of complete linkages (De Cáceres et al., 2015). Graph theory improves on these by avoiding assumptions concerning the cohesiveness of clusters and by focusing on neighbourhood interconnectivity rather than single pairwise resemblances or gradient length.

At least three advantages are apparent in the graph model. First, the method is not constrained by any fixed model of data structure; rather, it will accommodate data irrespective of the presence/absence of discontinuities and variability in cluster shape or density (Karypis et al., 1999). Second, for any given dataset, the degree to which any sample is interconnected with other samples in the graph is quantifiable from the graph structure, independent of any clustering solution. This feature can be leveraged to investigate the cause of classification instability because thresholds in certain graph metrics have been identified as outliers (Karypis, 2003). Third, in theory, graph partitioning should lead to more stable solutions because strong connections between samples within clusters should be robust to the addition of new samples.

In this paper, we assess the extent to which classification instability can be attributed to either deviation from the centroid (which quantifies noise when vegetation data are modelled on central‐tendency) or interconnectivity (which quantifies noise when vegetation data are modelled as graph networks). We simulated incremental changes in a classification arising from the addition of new data and hypothesised that:

1. The degree of individual sample interconnectivity (graph model) is a better indicator of instability (the future reallocation of that sample to a different group) than the degree of deviation from the centroid (central‐tendency model);
2. classification stability over multiple classification iterations is higher when undertaken using a graph model compared to a central‐tendency model;
3. the removal of outliers leads to more stable classifications from one iteration to the next; and
4. classification stability increases in successive iterations of the classification as the volume of data increases under either model (graph or central‐tendency).

In posing these hypotheses, we reasoned that a useful measure of the utility of any measure of noise is how well it forecasts instability in future iterations of the classification as new data are added, and that since the graph model is theoretically better suited to continuous data, it should produce more stable clustering solutions (hypotheses 1 and 2). We reasoned that if instability is a function of noisy samples continually changing groups, then the removal of those samples should increase classification stability (hypothesis 3). Finally, since our approach (described below) involved simulation of the development of a classification as progressively more data were added, we evaluated the evidence for the common assumption that clustering solutions become more stable with increasing sampling because ‘types’ become clear when represented by a sufficient number of samples (hypothesis 4).

## 2 METHODS

### 2.1 Approach

**FIGURE 1:**
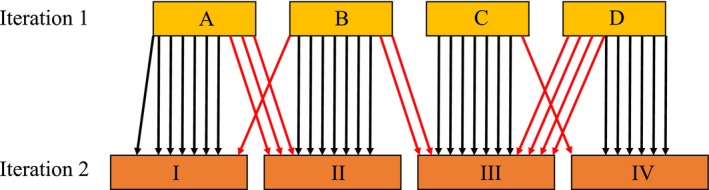
Conceptual basis for quantifying classification stability. A sample was categorised as stable if members of its cohort in the first iteration formed a relative majority in the second. Arrow colours represent samples that are either stable (black) or unstable (red) from one iteration to the next.

We simulated incremental changes in a classification arising from the addition of new data by performing clustering iteratively on progressively larger subsets of a large regional dataset comprising 7541 plots drawn from an area of 96,089 km^2^ located in southeast Australia. Our initial clustering solution was derived using a random selection of 50% of the available samples. This number approximated the number of samples available three decades ago (M. Tozer, unpubl. data), although our selection was almost certainly more evenly distributed in relation to environmental gradients than those available historically. We added a further random selection of 10% of the data (i.e. 20% of the remaining 50%) over five successive iterations in order to simulate the circumstances under which a classification is revised following the addition of new data. No fixed groups were involved; instead, new classes were allowed to form at each iteration. At each iteration, we categorised the classification of a sample as stable if members of its cluster in the first iteration formed a relative majority in its second (Figure 1). Stability was scored as the total number of stable samples divided by the number of samples clustered in the previous iteration. Relative majority has the disadvantage of being unable to discriminate between instability at the scale of individual samples (i.e. a sample leaving one cluster and joining another) and instability at the scale of clusters (i.e. a cluster dissolves into two or more sub‐clusters that, in turn, join other clusters). While both are important, only the former is expected to be predictable from individual sample metrics (distance to the centroid, interconnectivity). These scenarios are, however, the end points of a continuum of rearrangement outcomes, which are difficult to accommodate with simplicity because samples that relocate to different clusters usually do so in multiple blocs of variable size. Our study assumes, therefore, that solutions generated by the two methods have similar properties in this regard.

We compared the stability of classification between two algorithms predicated on contrasting models of data structure. We employed k‐means clustering (MacQueen, 1967), a centroid‐based technique, both because it is representative of approaches that model cluster membership on the basis of central‐tendency (proximity to group centroid) and because it has been widely applied in the classification of vegetation data (Kent, 2011). This method seeks compact, hyper‐spheroidal clusters (Aho et al., 2008) by minimising the sum of the squared distances between cluster members and the theoretical cluster centre point (centroid). The application of k‐means clustering in vegetation science is atypical in employing dissimilarity metrics instead of Euclidean distance in order to weight shared species presence over shared absence (Faith et al., 1987) and to specifically avoid anomalies in which samples with no species in common have higher similarity than samples that share species in common (Orlóci, 1978; Ricotta, 2019). That is, while centroids are calculated using species scores, plot‐to‐centroid distances are represented by non‐Euclidean dissimilarity metrics.

We chose graph partitioning as a representative of approaches that model interconnectivity (Karypis et al., 1999). This method has rarely been applied to the clustering of vegetation data; however, evidence is emerging that suggests graph partitioning has a number of key features integral to the successful retrieval of broad vegetation groups from plot data (Tozer & Keith, 2022). These include the capacity to adapt automatically to variability in data structure, high scalability and being non‐reliant on any fixed data model (Han et al., 2012). We employed a symmetric k‐nearest neighbours approach (Karypis, 2003). Under this model, a sparse graph is compiled in which samples are represented by vertices and edges exist between pairs of vertices when each vertex in the pair is included in the k‐nearest neighbours set of the other (in a symmetric model, no edge exists when one vertex is within the k‐nearest neighbours of the other but not vice versa). Since there are relatively few published applications from which to draw guidance on this method, we first undertook preliminary trials to test the effect of different parameter settings as described in Appendix S1.

### 2.2 Vegetation data

Vegetation data were obtained from the NSW Government BIONET data repository (DPE, 2022). We downloaded plot samples located within (or within 25 km of) the South East Highlands and Australian Alps Bioregions (SEWPaC, 2012) and applied pre‐determined filters to remove plots with low diversity of native species and/or high cover of exotic species (DPE, 2022), uncertain location or sampling areas other than the standard 0.04 ha, leaving 7541 plots. We removed naturalised species, taxa identified only at the generic level and standardised nomenclature according to DPE (2022). Cover‐abundance scores were transformed to presence data to eliminate differences among observers. The 7541 plots were assigned a random decimal number between zero and one (Excel version 16.64), from which we created five subsamples of the data corresponding to the 50th, 60th, 70th, 80th and 90th percentiles.

### 2.3 Clustering trials

We computed clustering solutions numbering 25, 50, 100, 150, 200 and 250 clusters from the complete dataset and five random selections of the data (i.e. 50% of samples and subsequent 10% increments). We analysed solutions across a range of cluster numbers (25–250) to determine the generality of our conclusions for applications requiring different levels of classification detail (our upper limit of 250 clusters approximates the number of units defined for the study area in the most recent classification exercise; DPE, 2022). We compared the attribution of samples to clusters across each of the five clustering iterations (ie successive solutions derived from 50% & 60%, 60% & 70%, 70% & 80%, 80% & 90% and 90% & 100% of the available data). For solutions of each cluster number, we computed the number of stable allocations as a proportion of the number of samples in the smaller of the two datasets and calculated an average (± standard deviation) over the five iterations.

K‐means solutions were generated using the ALOC module of the PATN package (Belbin, 1987) using the Bray & Curtis measure of association (Clarke, 1993) as adapted for presence–absence data (the Sörensen (‐Dice) index) and specifying random seeds with a maximum of 87 iterations and zero reallocations as stopping rules.

Graph partitioning was carried out using the scluster function in CLUTO software version 2.1.2 (Karypis, 2003), operating on a pairwise matrix of Bray and Curtis similarity scores (1—dissimilarity; Clarke, 1993). We first conducted preliminary trials on solutions of 25 and 250 clusters in order to determine appropriate specifications for edge weight and neighbourhood size for the construction of the sparse graph (Appendix S1). Edge weight refers to the strength of the link between pairs of vertices, and neighbourhood size determines the number of pairwise links (edges) constructed for each vertex (sample). Cluto employs hMetis (Karypis et al., 1999; Karypis & Kumar, 1998) to partition the k‐nearest neighbour graph into clusters by dissolving the edges between vertices. The algorithm targets the weakest edges and is guided by the objective of minimising the total edge cut (i.e. sum of edge weights). Based on our preliminary trials, we determined classification stability was relatively insensitive to the choice of either edge weight (pairwise similarity vs. number of samples common to each neighbourhood set) or the size of the neighbourhood sets. However, cluster solutions generated using a neighbourhood size proportional to the number of samples (prop) in combination with symmetric link (SL) edge weighting had marginally higher classification stability on average (Table S1), as reflected in a marginal significance test for the interaction term in analysis A (Table S2). We therefore adopted these parameters in all subsequent comparisons.

Cluto's scluster function allows for vertices with low interconnectivity to be eliminated from the graph prior to partitioning by specifying the minimum representation of the vertex in the member sets of its nearest neighbours. The reasoning is that those vertices not well represented in the neighbourhood sets of their nearest neighbours are likely to be outliers (Karypis, 2003). In addition, edges between vertices can be removed if their respective neighbourhood sets share a few vertices. The reasoning is that edges between vertices with fewer than the specified number of vertices in common are likely to bridge separate, independently interconnected clusters (Karypis, 2003). Our preliminary trials (Appendix S1) suggested that the number of vertices in common between a pair of samples connected by an edge is also an important indicator of outlier status, conveying information that is not quantified in the edge weight. We therefore applied vertex and edge pruning together, setting thresholds (*x*) at 0.1 and 0.2. That is, a vertex represented in fewer than (x*nnbrs) of the neighbourhood sets of its nearest neighbours was eliminated, where *nnbrs* = the number of nearest neighbours specified as a starting parameter. Similarly, an edge in the sparse graph was eliminated if fewer than (x*nnbrs) were common to the neighbour sets of both vertices.

### 2.4 Statistical analyses

**FIGURE 2:**
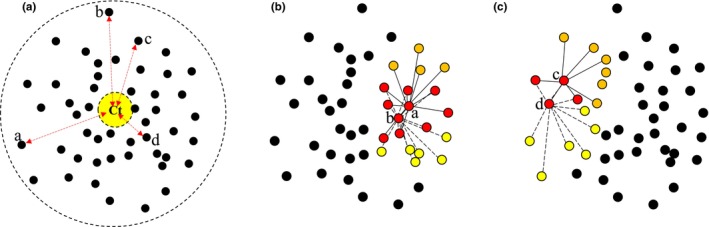
(a) Diagrammatic representation of deviation from centroid for samples a–d (length of red arrows) in relation to the yellow cluster centroid (*C*_t_). (b, c) Diagrammatic representation of different degrees of interconnectivity between samples a and b (b) and c and d (c). Red samples are included within the nearest neighbour member sets of both pairs. Orange samples are included in the member set of samples a and c, but not b and d. Yellow samples are included in the member set of samples b and d, but not a and c. There is a larger number of samples common to the member sets of a and b; therefore, they are more highly interconnected than samples c and d.

We used logistic regression (Table 1, H1) to evaluate the extent to which classification stability was a function of either (i) degree of deviation from centroid (k‐means) or (ii) interconnectivity (graph model) in cluster solutions of 25 categories using the generalised linear model function (glm) in the Stats package (R Core Team, 2021). Each sample (vertex) was treated as a replicate with a binary response (stable/unstable). For the k‐means model, the independent variable was the strength of cluster membership, represented by distance from the cluster centroid (Figure 2). This measure is not intrinsic to a sample within any data set, being specific to a particular clustering solution. For the graph model, the independent variable was the degree of interconnectivity (Figure 2). We quantified this value for each sample as the threshold at which pruning resulted in the sample's exclusion from the analysis. We computed sample interconnectivity independently for each sample in each data subset by progressively increasing the pruning threshold (vertex and edge in concert) with a fixed neighbour size of 100 samples. For example, a sample eliminated with the pruning threshold set at 0.2 was calculated to have interconnectivity = 0.2*100 (x*nnbrs). This measure is intrinsic for each sample in each data subset (i.e. independent of any clustering solution). We range‐standardised both metrics to facilitate the comparison of model coefficients (i.e. change in the log‐odds ratio for every unit change in deviation from centroid or interconnectivity metrics). Ten models were fitted, representing five clustering iterations for each of the two models. Model coefficients (mean model with logit link) were assessed using *z*‐tests.

**TABLE 1:** Summary of analyses undertaken in relation to hypotheses tested.

Analysis	Factors	Method	Response data
H1: Probability of future reallocation	Test each model independently Interconnectivity versus degree of deviation from centroid (k‐means)	Logistic regression	Binary (individual sample clustering is stable or not)
H2: Classification stability	Model (k‐means, graph) Number of clusters (25–250)	Beta regression	Proportion of samples with stable classification in each iteration
H3: Vertex and edge pruning (noise/outlier removal)	Number of clusters (25–250) Degree of pruning (0.1, 0.2)	Beta regression	Proportion of samples with stable classification in each iteration
H4: Data volume	Model (k‐means, graph) Iterations (as factor, 1–5) Number of clusters (25–250)	Beta regression	Proportion of samples with stable classification

We used beta regression (Cribari‐Neto & Zeileis, 2010) to test for differences in clustering stability related to model structure (central‐tendency vs. interconnectivity, Table 1, H2), the presence of outliers (noise) (graph partitioning only, H3) or the number of samples (Table 1, H4). We did not analyse the effects of removing outliers identified via the central‐tendency model because outlier status is not meaningful except where the centroids are ‘fixed’. Analyses were carried out using the Betareg package in R, which accommodates data that is not strictly independent (Cribari‐Neto & Zeileis, 2010). All analyses were carried out using R Version 1.4.1717 (R Core Team, 2021).

## 3 RESULTS

### 3.1 Hypothesis 1: Association between degree of deviation from centroid/interconnectivity and classification stability

**FIGURE 3:**
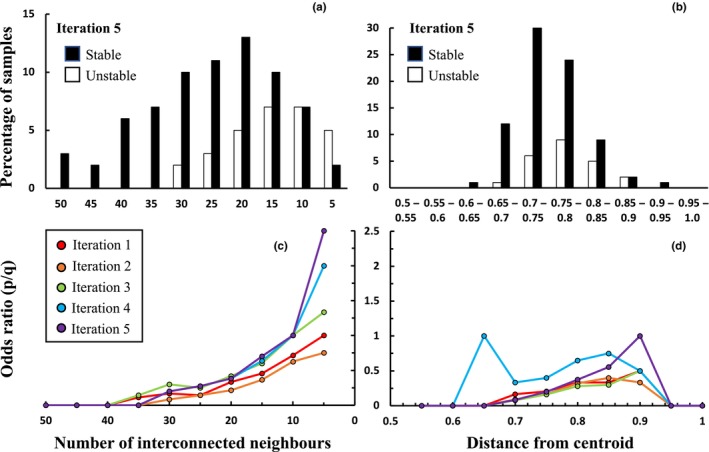
Percentage of samples stable (black columns)/unstable (white columns) from iteration four to five as a function of degree of interconnectivity (a) or degree of deviation from the centroid (b). (c, d) Represent the same data as odds ratios for all five iterations. Deviation from centroid values exhibited a high concentration around the median within the observed range (0.5–1).

**FIGURE 4:**
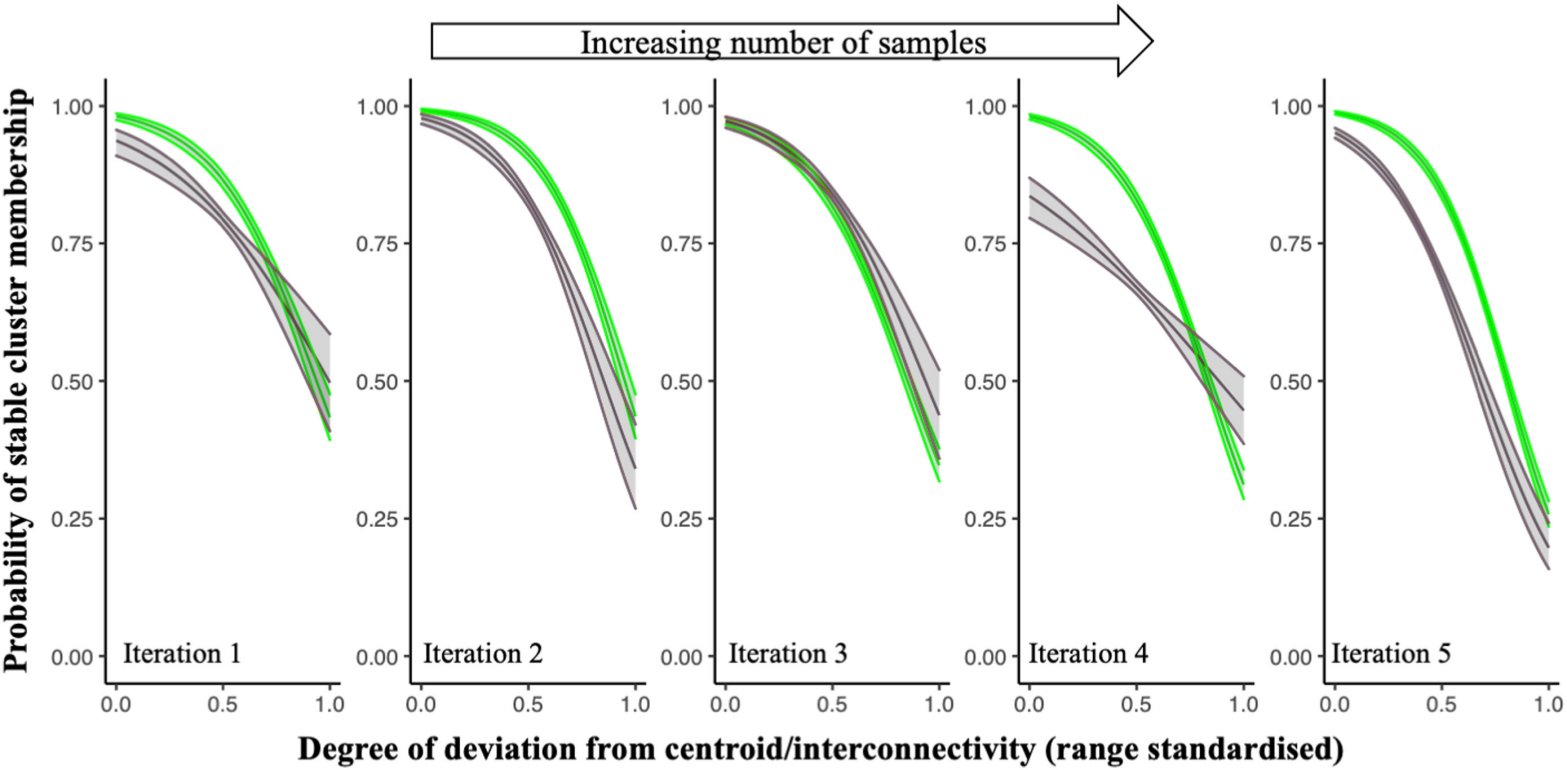
Probability that a sample remained stable in its group membership as a function of degree of interconnectivity (green) or degree of deviation from the centroid (grey) (95% confidence limits). The *x* axis is range‐standardised, with low values indicating high interconnectivity/deviation from the centroid.

Degree of interconnectivity was a superior indicator of the likelihood that a sample exhibited stability in clustering between iterations, accounting for on average five times (range 4–18 times) the deviance accounted for by degree of deviation from the centroid and with much smaller confidence bounds on the estimated coefficients (Table 2). Coefficients indicated a larger change in the log/odds ratio per unit change in degree of deviation from the centroid (central‐tendency model) compared with degree of interconnectivity (graph model). This reflects the strong inverse relationship between interconnectivity and log/odds (Figure 3a,c) (which flattens the logistic curve) compared with the weak and inconsistent trend associated with distance to the centroid (Figure 3b,d) (which causes a steep transition from high to low probability). Significant skews were evident in the distribution of stable/unstable samples in relation to the degree of interconnectivity but not the degree of deviation from the centroid (Figure 3, Appendix S2). The difference between stable and unstable samples became clearer with increasing numbers of samples in both measures, as reflected in lower probability values modelled at the end of the range (Figure 4).

**TABLE 2:** Logistic regression coefficients (change in the log‐odds ratio) and deviance change for models assessing the probability of a sample being classified consistently from one iteration to the next.

Model	Comparison (iteration)	Coefficient (2.5%) C (97.5%)	Deviance change (df)	AIC
Graph	S50–S60	(0.009) 0.014 (0.023)	416 (1)	3489
K‐means	S50–S60	(0.032) 0.066 (0.137)	53 (1)	3871
Graph	S60–S70	(0.003) 0.006 (0.009)	584 (1)	3704
K‐means	S60–S70	(0.006) 0.012 (0.024)	152 (1)	4259
Graph	S70–S80	(0.01) 0.014 (0.02)	712 (1)	5642
K‐means	S70–S80	(0.011) 0.022 (0.044)	122 (1)	4677
Graph	S80–S90	(0.006) 0.009 (0.012)	943 (1)	6332
K‐means	S80–S90	(0.095) 0.158 (0.262)	52 (1)	7666
Graph	S90–S100	(0.003) 0.004 (0.006)	1358 (1)	6925
K‐means	S90–S100	(0.008) 0.012 (0.019)	394 (1)	6930

### 3.2 Hypotheses 2: Are cluster solutions modelled on interconnectivity more consistent than those modelled on central‐tendency?

**FIGURE 5:**
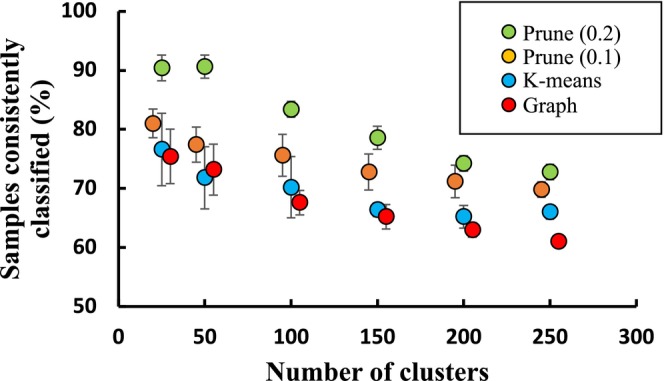
Classification stability as a function of the data model (central‐tendency vs. interconnectivity) and the extent to which weakly interconnected samples were pruned from the data before analysis. Note that the *x*‐values for the orange and red symbols were offset slightly below and above their actual values to avoid confusion caused by overlapping error bars.

No significant differences in classification stability were detected between k‐means clustering and graph partitioning when data were pooled across iterations (Table 3, Figure 5). Both models show declining stability with increasing numbers of clusters (Figure 5). In addition, while the members of stable groups almost always formed an absolute majority of the samples in their new group when the number of clusters in the solution was low, the proportion of stable groups comprising only a relative majority increased with the increasing number of clusters.

**TABLE 3:** Beta regression coefficients for models used to test for differences in clustering stability related to model structure (categorical variable: central‐tendency vs. interconnectivity [SL]), removal of weakly interconnected samples and volume of data.

Term	Estimate	Std. error	*Z* value	*p* > |*z*|
Hypothesis 2				
Intercept	0.91	0.081	11.25	<.001
Clusters	−0.001	0.001	−2.27	<.05
Model (SL)	0.122	0.114	1.07	ns (.287)
Clusters*Model (SL)	−0.001	0.001	−1.81	ns (.071)
Hypothesis 3				
Intercept	1.408	0.059	23.94	<.001
Clusters	−0.002	0.000	−6.67	<.001
Prune (0.2)	0.900	0.094	9.59	<.001
Prune (0)	−0.371	0.080	−4.66	<.001
Clusters*Prune (0.2)	−0.003	0.001	−5.91	<.001
Clusters*Prune (0)	−0.000	0.001	−0.12	ns (.902)
Hypothesis 4				
Intercept	0.956	0.135	7.11	<.001
Clusters	0.002	0.001	−2.29	<.05
Model (SL)	0.482	0.192	2.51	<.05
Iteration	0.053	0.042	1.30	ns (.195)
Clusters:Model (SL)	−0.003	0.001	−2.24	<.05
Clusters:Iteration	−0.0001	0.0001	−0.32	ns (.749)
Model (SL):Iteration	−0.152	0.0001	−2.63	<.01
Clusters:Model (SL):Iteration	0.001	0.0004	1.75	ns (.081)

### 3.3 Hypothesis 3: Does classification stability increase following the removal of weakly interconnected samples?

**FIGURE 6:**
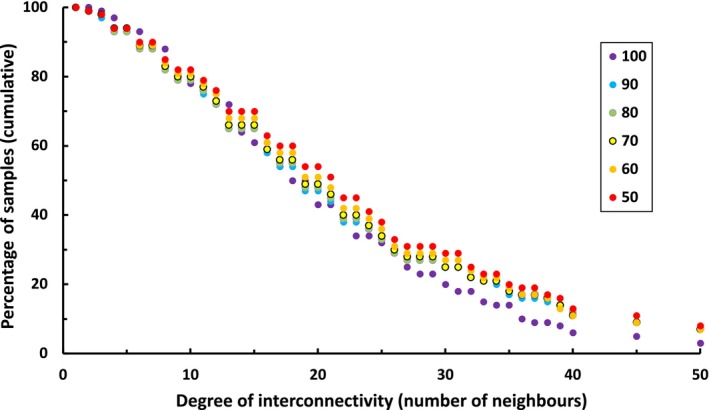
Distribution of samples in our dataset in relation to their degree of interconnectivity. Colours indicate the percentage of the available data included in the analysis.

Classification stability increased when vertex and edge pruning were applied in combination, with a higher threshold resulting in higher stability (Table 3, Figure 5). Stability declined with increasing numbers of clusters, as did the magnitude of the improvement attributable to pruning (Table 3, Figure 5). Increased classification stability by pruning was achieved at the cost of significant loss of information, with the application of pruning thresholds of 0.1 and 0.2 resulting in the removal of approximately 20% and 50% of the total data available in each subset (Figure 6).

### 3.4 Hypothesis 4: Relationships between classification stability and data volume

There was no evidence of increasing classification stability as the volume of data increased with each iteration (Table 3). A significant interactive effect (Clusters: Model [SL]) was detected, reflecting slightly lower stability in solutions of 250 clusters derived from the graph model compared with k‐means. There was a weak trend towards reduced interconnectivity across the data as the number of samples increased, with the full dataset showing a slightly larger skew in the number of weakly interconnected samples than any of the subsets (Figure 6).

## 4 DISCUSSION

### 4.1 Is interconnectivity a better indicator of instability?

Our results support our hypothesis that the degree of interconnectivity is a better indicator of classification stability than the degree of deviation from the centroid. Poorly interconnected samples had a much higher probability of changing classes between iterations, and conversely, well‐interconnected samples had a higher probability of remaining within their respective classification groups from one iteration to the next. In contrast, the degree to which samples deviated from centroids in one iteration had relatively little bearing on whether or not they changed groups, with the exception of a very small number of samples that either very strongly or very weakly resembled the centroid. As a consequence, interconnectivity explained a much higher proportion of deviance in the modelled outcome (sample changed class or not) compared to the degree of deviation from the centroid.

Our results are consistent with the concept of cluster mobility: distances to centroids are properties of clustering solutions rather than properties inherent in any data. Furthermore, they highlight the danger of overweighting the inferences drawn from clustering solutions and reinforcing them against later evidence that may not support them. As currently formulated, noise clustering promotes a clearer picture of patterns in vegetation data by differentiating between samples with strong resemblance to type and those with very little resemblance (De Cáceres et al., 2010; Wiser & De Cáceres, 2013). While this is useful for interpreting patterns within a particular iteration of a classification, our results suggest that the partitioning of samples between type and noise classes does not yield particularly strong insights into how the samples will be partitioned in subsequent iterations as more data are added. Since proximity to the centroid has little bearing on clustering stability, it cannot be assumed that the same set of samples constitutes noise in successive clustering of a growing data set. It is problematic, therefore, if the primary motivation of noise clustering and related approaches such as semi‐supervised clustering (Tichy et al., 2014) is seeking to ‘fix’ elements of a classification (Wiser & De Cáceres, 2013). From that perspective, the procedure is open to the criticism of essentialism (reification) (Keddy, 1993; Palmer & White, 1994; Wiegleb, 1989) and contrary to the consensus surrounding vegetation units as abstract entities, of which no stable classification is expected (Mucina, 1997).

### 4.2 Stability of graph‐based and central‐tendency classifications and the effect of removing outliers

Contrary to expectation, we did not find any difference in classification stability between clustering based on a graph model compared to a central‐tendency model, irrespective of the number of clusters created. The theoretical advantages of graph‐based clustering stem from its basis in raw pairwise dissimilarities that do not change when new data are added; hence, the basis for joint membership is constant from one iteration to the next. Samples that are strongly interconnected in one solution should therefore remain strongly interconnected, provided there are sufficient edges to accommodate new vertices as data are added. In contrast, we expected the assembly of clusters in central‐tendency methods to change when new data were added, effectively making the centroids mobile between successive iterations of classification.

We attribute the lack of observed differences in stability between methods to a very high proportion of weakly interconnected samples in our data (Figure 6). That is, we infer that the 20% of the samples in our data represented in the neighbour lists of 10 or fewer of their 100 nearest neighbours were not sufficiently interconnected to prevent the displacement of edges as new data were added. This interpretation is supported by our finding that the removal of samples with low interconnectivity increased the stability of the classification from one iteration to the next and that the more samples were removed, the more stable the classifications became (Table 3, Figure 5). This result is consistent with the notion that instability is caused by outlier samples because, in graph theory, outlier samples can be characterised as those with low representation in the sets of their nearest neighbours (Karypis, 2003).

The development of measures such as interconnectivity that are independent of any particular configuration of samples or types has the potential to improve the noise clustering model by reformulating it as a proactive procedure. That is, samples that are not amenable to classification within the context of a given dataset could be identified and omitted a priori from the classification while contributing to the design of gap‐fill sampling. Even so, as with noise clustering, some guidance is required to determine appropriate thresholds for omission, and metrics themselves provide no insight into the causes of noise. The exclusion of atypical samples therefore needs to be undertaken with care, balancing the potential improvements in classification stability with the risk of losing information. For example, atypical samples may reflect: (i) biases in the distribution of samples that result in under‐sampling of parts of environmental gradients (gaps); (ii) rare or extreme environments; or (iii) depleted or degraded vegetation types. Such samples may appear as outliers but nonetheless warrant a place in classifications.

Gaps can potentially be identified by analysing environmental gradients associated with species turnover and quantifying the distribution of samples relative to the expression of those environmental gradients in geographic space (Austin & Smith, 1989). Rare environments may always be, by nature, underrepresented in sample data and so their classification may require manual adjustments to clustering solutions. The treatment of depleted or degraded vegetation is more problematic. Mucina (1997) warned that the search for stability in syntaxonomic systems would always prove illusory if temporal factors such as past management and disturbance history were ignored. Historical perturbations are manifested as deviations from a starting configuration and vary in both the magnitude of deviation and the ensuing temporal trajectory (Bartha et al., 2008). By convention, assemblages resulting from extreme perturbations are placed outside the traditional syntaxonomic framework (Moravec, 1989) because they are idiosyncratic, unstable and follow unknown trajectories. However, extreme perturbations represent only one end of a spectrum of circumstances. Collectively, they are temporal gradients that cause fluctuations in species' abundance. Whether natural (e.g. seasonal fluctuations, decadal cycles of drought or flood) or anthropogenic in origin, processes that drive species loss or changes in detectability will inevitably introduce instability into classification systems based on similarity metrics because they are highly sensitive to such changes.

Our study provides some insights into the nature of instability in degraded or depleted vegetation types because the study area encompassed large areas of protected vegetation in topographically variable terrain as well as extensive grassy woodlands with a long history of intensive pastoralism that caused species loss and reduced detectability (Prober et al., 2017). We speculate that samples represented fewer than 10 times in the neighbour sets of their nearest neighbours plausibly constitute the outliers in our data, as this is the point at which the log/odds ratio first exceeded unity (sample re‐assignment becomes more likely than not, Figure 3c). Intuitively, one would expect the most heterogeneous groups to contain the most weakly interconnected samples, and these samples, in turn, represent vegetation that has been altered to various degrees by past disturbances. Indeed, in our dataset, the number of samples excluded by the application of our thresholds (10 and 20) corresponds approximately to the number of samples in the 10 and 50 most heterogenous types, respectively (Appendix S3). While further analyses are required to determine if this is the case, in many applications it would be undesirable to exclude these groups from analysis, not least because the number of samples removed is large (1500–3770 samples), especially considering the data were filtered prior to analysis to remove samples with an abnormally low richness of native species, a high abundance of exotic species or structural abnormalities. In comparison, a recent classification of the same data (DPE, 2022) resolved 258 vegetation types, with 1170 out of 7541 samples remaining unattributed to types (DPE, 2022). We conclude that the utility of measures of instability such as interconnectivity therefore lies not only in identifying samples that are not amenable to classification (outliers) but also in diagnosing unstable patterns in the data that require further interpretation.

### 4.3 Do vegetation classifications become more stable with increasing number of samples?

Although the capacity to predict which samples were unstable improved as the number of samples was progressively increased from 50% to 100% of the available data, we found no evidence of increasing classification stability. In theory, samples that were weakly interconnected in our initial dataset (50% of the available data) should have become more strongly interconnected as samples with similar compositions were added back in. In fact, although the distribution of samples was similar in the data used in each of our iterations, the full dataset showed a slightly larger skew in the number of weakly interconnected samples than any of the subsets, and there was a weak trend of increasing sample interconnectivity with a decreasing number of samples. Further investigations are required to determine whether this trend arose due to the chance of partitioning our data into subsamples or if it reflects some systematic properties of the data. We acknowledge that our simulations probably do not reflect the way in which our dataset was compiled through time, which is likely to have commenced opportunistically (targeting accessible areas) switching to gap‐filling over time. In theory, this could imbue a developing classification with greater stability if patterns in accessible areas were well sampled prior to switching to gap‐filling. However, the difficulties inherent in characterising a ‘typical’ pattern of data accumulation suggest any generalities emerging from this line of investigation may be limited. We also note that the intensity of sampling necessary to reliably elucidate vegetation patterns is unknown. Our samples comprise only 0.003% of the area of our study area, and it remains to be seen whether the patterns we observed in our simulations are sustained following increases in sampling over several orders of magnitude. Nevertheless, the contemporary distribution of samples in relation to degree of interconnectivity appears to support Mucina's (1997) contention that the search for stability through the addition of more data is futile because new data comprise both samples that resemble existing samples in the data and outliers, which, in our simulation, were approximately equal in proportion. For a well‐designed (environmentally & spatially stratified) sampling programme, new outliers may be expected to diminish proportionately over time.

Finally, the general trends in our results were consistent for solutions across the range of cluster numbers. However, both classification stability and the degree of improvement in stability achieved by the removal of outliers declined under both graph and central‐tendency models as the number of clusters in the solution increased (Figure 5). This pattern is consistent with our previous findings that, as the number of clusters increases, both k‐means and graph models place increasing proportions of samples in clusters that do not contain their nearest neighbours (Tozer & Keith, 2022). Such ‘misplacements’ should render a sample more sensitive to reallocation on subsequent iterations of analysis, and therefore classifications with larger numbers of types may be inherently more unstable than solutions with fewer types, assuming they are derived from the same data. This increasing instability at finer thematic resolution could be associated with ever finer partitioning of a continuum per se (i.e. fine partitions are inherently vague sensu; Regan et al., 2002), or putative types may simply be unstable until they can be defined by adequate numbers of samples.

## 5 CONCLUSION

Despite the consensus that vegetation is continuous but can be classified by convention (Keddy, 1993; Mucina, 1997; Palmer & White, 1994), there has apparently been little debate on how this should be done. The convention is founded on a logical proposition that non‐identical objects can be grouped on the basis of shared properties that differentiate them from members of other groups (Tüxen, 1955, cited in Moravec (1989)). While it does not follow that group membership should necessarily be determined with reference to average state, central‐tendency approaches have unarguably dominated vegetation science (Aho et al., 2008). In this paper, we have argued that for continuous patterns, the challenge of classification stability or robustness is better formulated as a question of how well a sample resembles other samples in the dataset (interconnectivity) rather than how well it resembles the average states of types in any particular clustering solution (central‐tendency). While both formulations have intuitive appeal, the former has the advantage of being untethered from any particular allocation of samples to types. Our results demonstrate that this feature unlocks the possibility of reducing classification instability proactively by removing outliers a priori, or at least a more systematic diagnosis of the nature of noise.

## AUTHOR CONTRIBUTIONS

**Mark G. Tozer:** Conceptualization (equal); data curation (lead); formal analysis (lead); methodology (lead); writing – original draft (lead); writing – review and editing (lead). **David A. Keith:** Conceptualization (equal); data curation (supporting); formal analysis (supporting); funding acquisition (lead); investigation (equal); methodology (supporting); project administration (lead); supervision (equal); writing – original draft (supporting); writing – review and editing (equal).

## FUNDING INFORMATION

This research was supported by an Australian Government Research Training Program scholarship.

## CONFLICT OF INTEREST STATEMENT

The authors declare no conflicts of interest exist in the presentation of this work.

## Supporting information


Appendix S1.

## Data Availability

CLUTO software modules are available for download from the Karypis Lab website (http://glaros.dtc.umn.edu/gkhome/cluto/cluto/download). A more recent implementation of the Chameleon algorithm in JAVA is included as a module in the clustering platform Clueminer (https://github.com/deric/clueminer). Plot data used in our analyses are available at: https://www.environment.nsw.gov.au/research/Vegetationinformationsystem.htm (NSW DPIE 2020, accessed 2nd August 2016). All analyses were performed on a matrix of similarity (1‐Bray–Curtis dissimilarity) between the objects to be clustered. Data were imported in a plain text file with *n* + 1 lines, with the first line containing the number of rows and the remaining n lines containing similarity values for each row (Karypis, 2003).
