# Supplementary material for: Beyond central‐tendency: If we agree discrete vegetation communities do not exist, should we investigate other methods of clustering?
Source: Ecol Evol. 2023 Nov 20;13(11):e10757. doi: 10.1002/ece3.10757 (PMC10659940; doi:10.1002/ece3.10757)
Supplement: Supplementary file 1 — Appendix S1. [file ECE3-13-e10757-s001.docx]

**Appendices**

**Appendix S1: preliminary trials to determine appropriate parameters for graph partitioning.**

Our preliminary trials aimed to determine whether clustering stability is affected: i) by how edge strength is modelled, and ii) how neighbour size is specified to accommodate increasing numbers of vertices. We evaluated two measures of edge weight. The first, direct link (sd, default setting), specified weight as the similarity between vertex pairs, a setting which effectively minimises the affinity between samples in different clusters (Karypis, Han and Kumar 1999). The second, symmetric link (sl), specified weight as the number of vertices common to the nearest-neighbour sets of each of the pair of vertices. This setting weights the extent to which sample pairs are members of interconnected sets over their direct connection (similarity).

We reasoned that clustering stability could be reduced if displacement of edges between interconnected vertices occurs when neighbourhood size is constrained while further samples are added. To investigate this, we undertook trials in which neighbourhood was either fixed (30 or 60 neighbours, reflecting appropriate lower and upper setting for datasets within this size range) or increased incrementally as data were added. We selected a neighbourhood size proportional (prop) the number of samples in the dataset, commencing at 30 neighbours for the initial trial (50% of the data) and increasing by 6 neighbours with each iteration up to a maximum of 60 for the complete dataset. The rationale was to allow interconnected sets to grow as more samples were added without the constraint a fixed neighbourhood size.

Cluto’s scluster function allows for vertices with low interconnectivity to be eliminated from the graph prior to partitioning by specifying the minimum representation of the vertex in the member sets of its nearest neighbours. The reasoning is those vertices not well represented in the neighbourhood-sets of its nearest neighbours are likely to be outliers (Karypis 2003). Alternatively, edges between vertices can be removed if their respective neighbourhood-sets share few vertices. The reasoning here is that edges between vertices with fewer than the specified number of vertices in common are likely to bridge separate, independently interconnected clusters (Karypis 2003). We repeated our trials to investigate whether classification stability was affected by the elimination of either outliers or bridging edges, applying vertex and edge pruning both independently and in concert. Thresholds (*x*) for these trials were set at 0.1 and 0.2. For example, a vertex represented in fewer than *(x * nnbrs)* of the neighbourhood sets of its nearest neighbours was illuminated, where *nnbrs* = the number of nearest neighbours as specified as a starting parameter.

*Statistical analyses*

We used beta regression (Cribari-Neto and Zeileis 2010) to test for differences in clustering stability related to algorithm parameters (graph-partitioning only), model structure (central-tendency vs interconnectivity) or volume of data (Table S1). Analyses were carried out using the Betareg package in Rstudio (Cribari-Neto & Zeileis, 2010).

Table S1.1 Summary of analyses undertaken in relation to hypotheses tested.

| Analysis | Factors | Method | Response |
| --- | --- | --- | --- |
| A) Model parameters (graph-partitioning only) | Edge weight (sd, sl)  Neighbourhood size (30, 60, prop) | Beta regression | proportion of samples classified consistently over 5 iterations |
| B) Pruning (graph-partitioning only) | Type (vertex, edge, both)  Degree of pruning (0.1, 0.2) | Beta regression | proportion of samples classified consistently over 5 iterations |
| C) Pruning (graph-partitioning only) | Number of clusters (25, 50, 100, 150, 200, 250)  Degree of pruning (0.1, 0.2) | Beta regression | proportion of samples classified consistently over 5 iterations |

**Results**

*Analysis A: Model parameters*

Classification stability was relatively insensitive to choice of either edge weight (pairwise similarity vs number of samples common to each neighbourhood set) or the size of neighbourhood sets (Tables 1, 2). However, cluster solutions generated using a neighbourhood size proportional to the number of samples (prop) in combination with symmetric link (SL) edge weighting tended to have marginally higher classification stability on average (Table 2) as reflected in a marginal significance test for the interaction term in analysis A (Table 3). We therefore adopted these parameters in all subsequent comparisons.

*Analyses B and C: Pruning*

Neither vertex nor edge pruning resulted in any significant improvement in classification stability when applied in isolation with a threshold of 0.1 (samples/edges represented in fewer than 10% of their neighbours member sets excluded). Classification stability increased when vertex and edge pruning were applied in combination, with a higher threshold resulting in higher stability (Tables 2, 3). Classification stability increased when edge pruning was applied using a threshold of 0.2 (Tables 2,3). Vertex and edge pruning in combination resulted in increased classification stability in clustering solutions spanning the range of cluster numbers, stability declined with increasing numbers of clusters as did the magnitude of the improved attributable to pruning (Table 3, Figure 3).

Table S1.2: Model coefficients for analyses A – C described in Table S1.

| Term | | Estimate | | Std. error | Z value | | | | P > \|z\| |  |  |
| --- | --- | --- | --- | --- | --- | --- | --- | --- | --- | --- | --- |
| 1. **Model parameters** | |  | |  |  | | | |  |  |  |
| Intercept | | 0.84 | | 0.06 | 13.13 | | | | < 0.001 |  |  |
| Edge wt (SL) | | -0.04 | | 0.09 | -0.44 | | | | ns (0.658) |  |  |
| Neigh. (60) | | 0.01 | | 0.09 | 0.06 | | | | ns (0.953) |  |  |
| Neigh. (prop) | | 0.1 | | 0.09 | 1.03 | | | | ns (0.303) |  |  |
| Edge wt (SL) * Neigh. (60) | | 0.16 | | 0.13 | 1.22 | | | | ns (0.222) |  |  |
| Edge wt (SL) * Neigh. (prop) | | 0.26 | | 0.13 | 1.96 | | | | ns (0.050) |  |  |
| 1. **Pruning** | |  | |  |  | | | |  |  |  |
| Intercept | 0.72 | | 0.20 | | 3.66 | | < 0.001 | | | |  |
| Prune type € | -0.12 | | 0.27 | | -0.46 | | ns (0.649) | | | |  |
| Prune type (V) | 0.40 | | 0.26 | | 1.53 | | ns (0.127) | | | |  |
| Prune mag. (0.2) | 0.72 | | 0.14 | | 5.35 | | < 0.001 | | | |  |
| Prune type € * Prune mag. (0.2) | -0.15 | | 0.18 | | -0.82 | | ns (0.415) | | | |  |
| Prune type (V) * Prune mag. (0.2) | -0.71 | | 0.17 | | -4.12 | | < 0.001 | | | |  |
| 1. **Pruning** | |  | |  |  | | | |  |  |  |
| Intercept | | 1.408 | | 0.059 | | 23.94 | | < 0.001 | | | |
| Clusters | | -0.002 | | 0.000 | | -6.67 | | < 0.001 | | | |
| Prune (0.2) | | 0.900 | | 0.094 | | 9.59 | | < 0.001 | | | |
| Prune (0) | | -0.371 | | 0.080 | | -4.66 | | < 0.001 | | | |
| Clusters * Prune (0.2) | | -0.003 | | 0.001 | | -5.91 | | < 0.001 | | | |
| Clusters * Prune (0) | | -0.000 | | 0.001 | | -0.12 | | ns (0.902) | | | |

Table S1.3: Results of preliminary graph-partitioning trials used to determine appropriate parameters for stability of classification. Data are average stability recorded over five iterations for solutions of 25 and 250 clusters. Edge weight was varied between pairwise similarity (direct) and number of times a vertex was listed in the neighbourhood sets of its 50 nearest neighbours (link). Neighbour size was either fixed (30 or 60 neighbours) or varied in proportion to the number of samples clustered (prop). Pruning threshold were applied to vertices (V), edges € or both.

| Clusters | Edge weight | Neighbours | Prune | Stability (% + sd) |
| --- | --- | --- | --- | --- |
| 25 | direct | 30 | none | 70 (3.7) |
| 25 | link | 30 | none | 69 (2.9) |
| 25 | direct | 60 | none | 70 (1.9) |
| 25 | link | 60 | none | 73 (2.5) |
| 25 | direct | prop | none | 72 (2.8) |
| 25 | link | prop | none | 76 (4.9) |
| 25 | link | prop | V (0.1) | 76 (3.6) |
| 25 | link | prop | E (0.1) | 76 (3.8) |
| 25 | link | prop | V + E (0.1) | 81 (2.4) |
| 25 | link | prop | V (0.2) | 76 (3.7) |
| 25 | link | prop | E (0.2) | 85 (3.3) |
| 25 | link | prop | V + E (0.2) | 90 (2.2) |
| 250 | direct | 30 | none | 61 (2.1) |
| 250 | link | 30 | none | 63 (3.5) |
| 250 | direct | 60 | none | 56 (2.5) |
| 250 | link | 60 | none | 59 (3.3) |
| 250 | direct | prop | none | 59 (1.9) |
| 250 | link | prop | none | 61 (0.7) |
| 250 | link | prop | V + E (0.1) | 70 (1.3) |
| 250 | link | prop | V + E (0.2) | 73 (1.3) |

Table S1.4: Test different prune types and magnitude

Type and scale and interaction significant at P <0.001.

Log-likelihood: 64.65 on 7 Df

Pseudo R-squared: 0.8061

| Term | Estimate | Std. error | Z value | P > \|z\| |
| --- | --- | --- | --- | --- |
| Intercept | 0.72 | 0.20 | 3.66 | < 0.001 |
| Prune type € | -0.12 | 0.27 | -0.46 | ns (0.649) |
| Prune type (V) | 0.40 | 0.26 | 1.53 | ns (0.127) |
| Prune mag. (0.2) | 0.72 | 0.14 | 5.35 | < 0.001 |
| Prune type € * Prune mag. (0.2) | -0.15 | 0.18 | -0.82 | ns (0.415) |
| Prune type (V) * Prune mag. (0.2) | -0.71 | 0.17 | -4.12 | < 0.001 |

Table S1.5: Test number of clusters, neighbourhood size

Clusters significant at P <0.001, neighbours ns (p=0.347), interaction (p=0.070)

Log-likelihood: 94.91 on 5 Df

Pseudo R-squared: 0.3277

| Term | Estimate | Std. error | Z value | P > \|z\| |
| --- | --- | --- | --- | --- |
| Intercept | 0.91 | 0.081 | 11.25 | < 0.001 |
| Clusters | -0.001 | 0.001 | -2.27 | < 0.05 |
| Model (SL) | 0.122 | 0.114 | 1.07 | ns (0.287) |
| Clusters * Model (SL) | -0.001 | 0.001 | -1.81 | ns (0.071) |

Table S1.6: Test number of clusters, prune type

Main effects and interactions significant

Clusters significant at P <0.001, neighbours ns (p=0.347), interaction (p=0.070)

Log-likelihood: 189 on 7 Df

Pseudo R-squared: 0.8829

| Term | Estimate | Std. error | Z value | P > \|z\| |
| --- | --- | --- | --- | --- |
| Intercept | 1.408 | 0.059 | 23.94 | < 0.001 |
| Clusters | -0.002 | 0.000 | -6.67 | < 0.001 |
| Prune (0.2) | 0.900 | 0.094 | 9.59 | < 0.001 |
| Prune (0) | -0.371 | 0.080 | -4.66 | < 0.001 |
| Clusters * Prune (0.2) | -0.003 | 0.001 | -5.91 | < 0.001 |
| Clusters * Prune (0) | -0.000 | 0.001 | -0.12 | ns (0.902) |

Appendix S2

Figure S2.1: Percentage of samples classified consistently at each iteration (black columns) compared with the percentages reallocated to a new group (open bars) as a function of degree of interconnection (graph model, left column) of strength of cluster membership (central -tendency, distance to centroid). The distribution of degree of cluster membership values exhibited a higher degree of concentration around the median values within the observed range (0.5 – 1).

**Appendix S3**

**Table S3.1 Scluster metrics for 250 cluster solution with no pruning listing cluster sizes (column 2) in decreasing order of cluster homogeneity (column 3).**

Solution ---------------------------------------------------------------------

cid Size ISim ISdev ESim ESdev |

------------------------------------------------------------------------

0 7 +54.619 +0.932 +0.322 +0.008 |

1 6 +52.267 +1.468 +0.314 +0.013 |

2 13 +46.154 +1.646 +0.199 +0.010 |

3 8 +48.250 +2.289 +0.282 +0.027 |

4 12 +45.758 +1.565 +0.204 +0.013 |

5 16 +42.342 +2.749 +0.189 +0.043 |

6 7 +45.952 +0.975 +0.229 +0.009 |

7 7 +45.762 +1.554 +0.225 +0.013 |

8 4 +52.167 +1.575 +0.335 +0.011 |

9 7 +44.190 +0.935 +0.190 +0.007 |

10 12 +40.955 +2.069 +0.171 +0.019 |

11 9 +41.917 +1.122 +0.232 +0.022 |

12 12 +40.379 +1.677 +0.142 +0.011 |

13 5 +46.300 +0.512 +0.236 +0.008 |

14 13 +38.962 +2.111 +0.154 +0.012 |

15 12 +38.606 +1.958 +0.152 +0.015 |

16 15 +37.371 +1.795 +0.125 +0.021 |

17 7 +40.429 +1.863 +0.205 +0.013 |

18 3 +52.000 +1.323 +0.336 +0.019 |

19 5 +42.800 +1.217 +0.226 +0.012 |

20 4 +45.500 +2.728 +0.240 +0.011 |

21 6 +39.800 +2.363 +0.199 +0.026 |

22 6 +39.733 +2.096 +0.217 +0.020 |

23 8 +37.643 +2.691 +0.154 +0.026 |

24 7 +37.524 +0.974 +0.196 +0.012 |

25 6 +38.667 +1.033 +0.162 +0.016 |

26 6 +38.333 +1.695 +0.170 +0.011 |

27 14 +34.407 +2.593 +0.123 +0.019 |

28 3 +47.667 +1.041 +0.244 +0.002 |

29 15 +33.448 +2.958 +0.103 +0.021 |

30 9 +34.889 +1.384 +0.157 +0.028 |

31 3 +46.000 +0.500 +0.307 +0.003 |

32 4 +39.833 +0.577 +0.191 +0.008 |

33 10 +32.911 +2.449 +0.183 +0.015 |

34 10 +32.689 +3.997 +0.159 +0.027 |

35 15 +30.981 +3.038 +0.104 +0.025 |

36 4 +38.167 +1.171 +0.165 +0.011 |

37 9 +32.167 +1.259 +0.120 +0.015 |

38 14 +30.549 +2.373 +0.095 +0.018 |

39 5 +35.300 +2.381 +0.171 +0.021 |

40 3 +42.000 +0.500 +0.204 +0.008 |

41 8 +31.893 +9.016 +0.202 +0.021 |

42 6 +33.000 +2.266 +0.136 +0.025 |

43 13 +29.308 +2.792 +0.102 +0.017 |

44 6 +32.200 +4.420 +0.153 +0.025 |

45 4 +35.667 +3.018 +0.192 +0.025 |

46 8 +30.393 +4.236 +0.149 +0.025 |

47 4 +35.167 +2.701 +0.188 +0.024 |

48 12 +28.606 +8.247 +0.133 +0.027 |

49 7 +30.238 +3.131 +0.138 +0.022 |

50 6 +30.067 +2.145 +0.157 +0.032 |

51 3 +37.000 +1.500 +0.206 +0.007 |

52 12 +26.697 +5.990 +0.157 +0.036 |

53 5 +29.500 +2.129 +0.161 +0.018 |

54 6 +27.800 +1.043 +0.119 +0.017 |

55 13 +24.872 +6.071 +0.145 +0.017 |

56 12 +24.197 +7.143 +0.148 +0.040 |

57 7 +25.333 +5.982 +0.139 +0.015 |

58 9 +24.389 +6.803 +0.123 +0.031 |

59 13 +23.167 +6.833 +0.159 +0.026 |

60 7 +24.714 +3.582 +0.116 +0.014 |

61 6 +24.867 +6.113 +0.135 +0.033 |

62 18 +21.647 +4.422 +0.077 +0.014 |

63 12 +21.818 +7.759 +0.107 +0.025 |

64 31 +20.555 +6.586 +0.023 +0.017 |

65 23 +20.502 +10.031 +0.118 +0.052 |

66 10 +21.867 +5.404 +0.123 +0.023 |

67 17 +20.647 +4.662 +0.070 +0.032 |

68 6 +23.133 +3.527 +0.120 +0.019 |

69 12 +20.758 +6.198 +0.109 +0.029 |

70 24 +19.746 +4.747 +0.036 +0.015 |

71 20 +19.816 +5.043 +0.077 +0.016 |

72 5 +22.800 +3.768 +0.120 +0.020 |

73 9 +20.222 +6.105 +0.095 +0.015 |

74 24 +18.634 +6.209 +0.037 +0.016 |

75 7 +20.857 +4.327 +0.111 +0.023 |

76 19 +18.801 +3.723 +0.065 +0.018 |

77 21 +18.000 +4.296 +0.057 +0.020 |

78 8 +19.250 +4.892 +0.111 +0.023 |

79 8 +18.857 +3.075 +0.069 +0.016 |

80 8 +18.679 +3.289 +0.100 +0.018 |

81 7 +18.905 +2.961 +0.099 +0.019 |

82 24 +16.703 +7.990 +0.084 +0.032 |

83 11 +17.473 +4.133 +0.082 +0.018 |

84 18 +16.824 +5.905 +0.086 +0.029 |

85 12 +17.197 +5.563 +0.090 +0.016 |

86 9 +17.722 +4.587 +0.074 +0.020 |

87 16 +16.633 +5.137 +0.120 +0.023 |

88 14 +16.440 +6.612 +0.094 +0.035 |

89 27 +15.764 +4.576 +0.049 +0.017 |

90 22 +15.524 +7.077 +0.065 +0.024 |

91 14 +15.890 +5.550 +0.128 +0.042 |

92 7 +17.048 +4.096 +0.070 +0.015 |

93 8 +16.536 +5.067 +0.089 +0.030 |

94 17 +15.147 +4.662 +0.081 +0.027 |

95 10 +15.844 +4.733 +0.094 +0.026 |

96 33 +14.557 +6.210 +0.041 +0.019 |

97 43 +14.101 +7.098 +0.025 +0.020 |

98 19 +13.895 +5.479 +0.076 +0.020 |

99 7 +15.095 +5.229 +0.088 +0.017 |

100 10 +14.133 +4.254 +0.055 +0.015 |

101 28 +13.169 +4.289 +0.054 +0.026 |

102 35 +12.866 +5.921 +0.017 +0.014 |

103 14 +13.516 +5.258 +0.064 +0.026 |

104 19 +13.099 +6.187 +0.029 +0.019 |

105 10 +13.733 +4.702 +0.093 +0.018 |

106 29 +12.266 +4.143 +0.046 +0.017 |

107 27 +12.291 +5.904 +0.044 +0.018 |

108 10 +12.933 +3.799 +0.071 +0.021 |

109 20 +11.995 +4.813 +0.058 +0.023 |

110 12 +12.424 +4.223 +0.071 +0.022 |

111 23 +11.854 +4.589 +0.106 +0.050 |

112 16 +11.583 +4.611 +0.083 +0.024 |

113 12 +11.742 +4.954 +0.069 +0.026 |

114 9 +11.833 +5.907 +0.067 +0.020 |

115 19 +10.608 +5.082 +0.059 +0.026 |

116 33 +9.992 +5.058 +0.030 +0.018 |

117 12 +10.561 +4.160 +0.059 +0.019 |

118 20 +9.763 +3.992 +0.040 +0.019 |

119 13 +9.897 +6.890 +0.058 +0.035 |

120 16 +9.625 +4.263 +0.052 +0.024 |

121 12 +9.758 +3.905 +0.059 +0.014 |

122 52 +9.055 +4.897 +0.039 +0.019 |

123 15 +9.495 +3.011 +0.064 +0.016 |

124 42 +8.935 +4.477 +0.013 +0.009 |

125 25 +9.000 +3.697 +0.024 +0.014 |

126 38 +8.767 +4.293 +0.018 +0.013 |

127 20 +8.842 +4.929 +0.060 +0.016 |

128 20 +8.758 +4.074 +0.053 +0.012 |

129 10 +8.911 +3.600 +0.088 +0.028 |

130 12 +8.606 +4.750 +0.086 +0.044 |

131 9 +8.778 +1.999 +0.061 +0.014 |

132 13 +8.167 +2.793 +0.070 +0.023 |

133 18 +7.915 +3.893 +0.103 +0.017 |

134 14 +7.912 +3.995 +0.088 +0.034 |

135 22 +7.602 +4.943 +0.017 +0.015 |

136 28 +7.405 +4.204 +0.051 +0.022 |

137 14 +7.549 +3.677 +0.072 +0.023 |

138 14 +7.374 +4.258 +0.061 +0.029 |

139 32 +7.034 +4.851 +0.020 +0.018 |

140 24 +7.051 +3.814 +0.045 +0.021 |

141 12 +7.364 +3.020 +0.092 +0.034 |

142 20 +7.063 +4.571 +0.068 +0.029 |

143 50 +6.787 +4.873 +0.030 +0.023 |

144 10 +7.267 +3.602 +0.031 +0.014 |

145 26 +6.828 +2.928 +0.064 +0.013 |

146 33 +6.610 +3.013 +0.044 +0.024 |

147 12 +6.909 +5.561 +0.040 +0.021 |

148 58 +6.301 +4.422 +0.008 +0.009 |

149 8 +7.107 +3.257 +0.065 +0.025 |

150 14 +6.626 +2.925 +0.068 +0.024 |

151 19 +6.287 +2.022 +0.091 +0.034 |

152 35 +5.983 +3.401 +0.052 +0.027 |

153 38 +5.940 +3.871 +0.021 +0.019 |

154 21 +6.052 +4.120 +0.027 +0.018 |

155 27 +5.872 +3.174 +0.022 +0.015 |

156 15 +5.952 +3.521 +0.059 +0.021 |

157 17 +5.882 +2.884 +0.032 +0.015 |

158 23 +5.593 +4.335 +0.030 +0.023 |

159 9 +5.972 +3.824 +0.072 +0.027 |

160 24 +5.442 +2.833 +0.060 +0.031 |

161 73 +5.213 +4.050 +0.020 +0.015 |

162 14 +5.429 +3.318 +0.038 +0.017 |

163 52 +5.081 +3.100 +0.010 +0.011 |

164 36 +5.078 +3.376 +0.023 +0.015 |

165 10 +5.444 +3.154 +0.055 +0.018 |

166 16 +5.000 +3.472 +0.022 +0.010 |

167 86 +4.488 +3.303 +0.018 +0.014 |

168 28 +4.553 +2.914 +0.023 +0.016 |

169 24 +4.540 +3.305 +0.066 +0.028 |

170 36 +4.190 +2.704 +0.033 +0.020 |

171 59 +4.112 +2.900 +0.013 +0.011 |

172 15 +4.324 +2.991 +0.032 +0.020 |

173 60 +4.045 +3.601 +0.003 +0.004 |

174 30 +4.048 +3.302 +0.027 +0.022 |

175 41 +3.557 +1.968 +0.036 +0.014 |

176 15 +3.714 +2.041 +0.038 +0.019 |

177 30 +3.526 +2.531 +0.031 +0.018 |

178 16 +3.233 +1.810 +0.053 +0.025 |

179 67 +2.997 +2.129 +0.009 +0.006 |

180 25 +3.033 +1.615 +0.033 +0.013 |

181 27 +2.980 +2.524 +0.011 +0.010 |

182 43 +2.914 +1.810 +0.031 +0.016 |

183 33 +2.943 +1.940 +0.033 +0.013 |

184 24 +2.928 +1.671 +0.007 +0.005 |

185 14 +2.890 +1.269 +0.043 +0.020 |

186 147 +2.597 +2.043 +0.020 +0.016 |

187 17 +2.713 +1.740 +0.032 +0.013 |

188 13 +2.744 +1.654 +0.036 +0.016 |

189 18 +2.641 +1.993 +0.041 +0.031 |

190 21 +2.605 +1.500 +0.012 +0.004 |

191 85 +2.501 +1.685 +0.021 +0.012 |

192 49 +2.465 +1.866 +0.013 +0.010 |

193 24 +2.496 +1.713 +0.029 +0.011 |

194 24 +2.442 +1.522 +0.013 +0.011 |

195 33 +2.424 +1.675 +0.021 +0.014 |

196 32 +2.308 +1.835 +0.024 +0.019 |

197 76 +2.241 +2.138 +0.008 +0.009 |

198 128 +2.231 +2.044 +0.019 +0.019 |

199 65 +2.141 +1.503 +0.014 +0.010 |

200 17 +2.213 +1.287 +0.029 +0.014 |

201 62 +2.086 +2.086 +0.009 +0.009 |

202 29 +2.111 +1.827 +0.014 +0.010 |

203 29 +2.106 +1.400 +0.031 +0.011 |

204 39 +2.061 +1.345 +0.030 +0.012 |

205 18 +2.033 +1.905 +0.022 +0.012 |

206 17 +2.022 +0.932 +0.037 +0.017 |

207 16 +2.017 +1.093 +0.049 +0.032 |

208 75 +1.842 +2.123 +0.008 +0.007 |

209 69 +1.821 +1.492 +0.012 +0.010 |

210 54 +1.819 +1.219 +0.011 +0.012 |

211 53 +1.789 +1.179 +0.031 +0.019 |

212 108 +1.701 +1.452 +0.008 +0.009 |

213 75 +1.679 +1.487 +0.029 +0.016 |

214 41 +1.549 +1.239 +0.029 +0.018 |

215 36 +1.551 +1.184 +0.014 +0.012 |

216 46 +1.474 +1.095 +0.012 +0.009 |

217 108 +1.432 +1.961 +0.002 +0.001 |

218 66 +1.456 +1.459 +0.010 +0.011 |

219 57 +1.442 +1.131 +0.011 +0.006 |

220 60 +1.421 +1.220 +0.010 +0.009 |

221 60 +1.421 +1.299 +0.012 +0.010 |

222 28 +1.460 +1.035 +0.035 +0.021 |

223 13 +1.449 +0.852 +0.021 +0.008 |

224 107 +1.301 +1.286 +0.012 +0.011 |

225 73 +1.298 +1.273 +0.017 +0.014 |

226 83 +1.286 +1.140 +0.015 +0.010 |

227 32 +1.258 +1.182 +0.013 +0.012 |

228 95 +1.217 +1.340 +0.009 +0.007 |

229 22 +1.264 +0.819 +0.032 +0.015 |

230 47 +1.136 +1.125 +0.019 +0.014 |

231 199 +1.046 +1.268 +0.012 +0.014 |

232 35 +1.030 +0.732 +0.005 +0.006 |

233 80 +1.028 +0.839 +0.015 +0.013 |

234 31 +0.996 +0.963 +0.012 +0.010 |

235 34 +1.009 +0.551 +0.021 +0.013 |

236 98 +0.946 +1.021 +0.014 +0.011 |

237 100 +0.875 +0.888 +0.005 +0.007 |

238 190 +0.874 +0.978 +0.008 +0.009 |

239 55 +0.871 +0.652 +0.005 +0.003 |

240 140 +0.866 +0.781 +0.010 +0.010 |

241 117 +0.818 +0.753 +0.016 +0.011 |

242 49 +0.655 +0.595 +0.011 +0.010 |

243 40 +0.588 +0.398 +0.015 +0.010 |

244 65 +0.501 +0.484 +0.005 +0.004 |

245 64 +0.485 +0.573 +0.006 +0.007 |

246 78 +0.418 +0.316 +0.020 +0.015 |

247 61 +0.354 +0.327 +0.003 +0.004 |

248 299 +0.356 +0.354 +0.010 +0.010 |

249 161 +0.217 +0.264 +0.003 +0.003 |

**Table S3.2 Scluster metrics for 250 cluster solution with edge and vertex pruning set at a threshold of 0.1 listing cluster sizes (column 2) in decreasing order of cluster homogeneity (column 3).**

Solution ---------------------------------------------------------------------

cid Size ISim ISdev ESim ESdev |

------------------------------------------------------------------------

0 7 +54.476 +1.025 +0.427 +0.010 |

1 6 +51.800 +1.290 +0.415 +0.018 |

2 12 +46.182 +1.663 +0.271 +0.018 |

3 9 +46.944 +1.523 +0.299 +0.017 |

4 9 +45.694 +2.465 +0.331 +0.041 |

5 9 +45.389 +1.624 +0.289 +0.010 |

6 8 +45.964 +0.896 +0.364 +0.012 |

7 8 +45.321 +1.646 +0.358 +0.037 |

8 12 +42.667 +1.067 +0.210 +0.009 |

9 12 +42.000 +2.341 +0.245 +0.029 |

10 4 +51.167 +0.694 +0.445 +0.012 |

11 7 +43.429 +1.542 +0.274 +0.020 |

12 12 +40.379 +1.677 +0.188 +0.015 |

13 3 +54.333 +0.289 +0.460 +0.002 |

14 7 +41.810 +1.862 +0.281 +0.022 |

15 14 +38.077 +4.188 +0.221 +0.026 |

16 13 +38.218 +1.683 +0.183 +0.025 |

17 7 +41.190 +1.103 +0.232 +0.027 |

18 4 +45.667 +1.122 +0.315 +0.013 |

19 6 +40.933 +1.440 +0.279 +0.020 |

20 4 +44.667 +0.720 +0.321 +0.015 |

21 7 +38.524 +1.060 +0.267 +0.027 |

22 3 +49.333 +0.577 +0.319 +0.009 |

23 6 +38.800 +1.193 +0.235 +0.017 |

24 9 +36.250 +3.745 +0.249 +0.022 |

25 6 +38.667 +1.033 +0.215 +0.021 |

26 6 +38.600 +3.535 +0.250 +0.032 |

27 9 +35.722 +3.144 +0.242 +0.028 |

28 6 +37.800 +2.086 +0.285 +0.024 |

29 4 +41.667 +0.720 +0.295 +0.024 |

30 16 +32.933 +2.593 +0.142 +0.029 |

31 6 +36.800 +2.132 +0.252 +0.040 |

32 4 +40.500 +0.694 +0.254 +0.006 |

33 3 +45.667 +0.764 +0.322 +0.005 |

34 9 +34.083 +4.334 +0.194 +0.025 |

35 3 +44.667 +1.443 +0.351 +0.019 |

36 4 +38.667 +0.471 +0.249 +0.024 |

37 4 +38.167 +1.171 +0.219 +0.015 |

38 18 +30.248 +1.491 +0.073 +0.015 |

39 9 +32.167 +1.259 +0.159 +0.020 |

40 14 +30.736 +2.980 +0.141 +0.035 |

41 14 +30.549 +2.373 +0.125 +0.024 |

42 5 +35.400 +1.773 +0.247 +0.029 |

43 4 +37.500 +1.401 +0.237 +0.029 |

44 5 +33.900 +1.084 +0.179 +0.029 |

45 9 +29.833 +4.515 +0.216 +0.045 |

46 3 +39.667 +0.289 +0.278 +0.019 |

47 3 +39.333 +1.258 +0.273 +0.019 |

48 6 +31.267 +3.006 +0.205 +0.035 |

49 3 +38.000 +0.500 +0.255 +0.039 |

50 4 +33.167 +1.291 +0.254 +0.035 |

51 6 +29.533 +2.145 +0.176 +0.043 |

52 5 +30.800 +1.328 +0.226 +0.025 |

53 13 +26.436 +6.910 +0.188 +0.037 |

54 18 +25.634 +6.086 +0.130 +0.041 |

55 3 +36.000 +1.000 +0.301 +0.041 |

56 13 +24.436 +3.604 +0.117 +0.033 |

57 7 +26.238 +6.661 +0.176 +0.038 |

58 12 +24.455 +5.073 +0.202 +0.022 |

59 7 +26.048 +3.184 +0.141 +0.022 |

60 16 +23.542 +3.840 +0.113 +0.021 |

61 8 +25.071 +4.118 +0.176 +0.024 |

62 22 +22.649 +4.889 +0.089 +0.023 |

63 8 +23.929 +5.032 +0.128 +0.020 |

64 8 +23.250 +5.946 +0.156 +0.036 |

65 8 +23.179 +6.570 +0.209 +0.042 |

66 16 +21.575 +6.458 +0.127 +0.037 |

67 6 +24.200 +5.007 +0.177 +0.054 |

68 17 +21.338 +3.508 +0.093 +0.019 |

69 24 +20.938 +5.107 +0.081 +0.034 |

70 13 +21.603 +6.823 +0.189 +0.049 |

71 5 +24.800 +5.206 +0.157 +0.036 |

72 6 +23.133 +3.527 +0.159 +0.025 |

73 7 +22.095 +4.689 +0.135 +0.028 |

74 9 +21.056 +4.571 +0.138 +0.023 |

75 17 +19.331 +6.281 +0.152 +0.071 |

76 10 +19.800 +6.045 +0.154 +0.045 |

77 13 +18.641 +6.783 +0.197 +0.041 |

78 12 +18.561 +6.389 +0.131 +0.028 |

79 12 +18.182 +5.857 +0.109 +0.047 |

80 13 +17.795 +7.252 +0.134 +0.041 |

81 11 +17.927 +5.077 +0.126 +0.034 |

82 13 +17.423 +8.289 +0.131 +0.075 |

83 7 +18.190 +3.613 +0.120 +0.037 |

84 12 +16.424 +5.367 +0.075 +0.026 |

85 6 +17.667 +4.992 +0.103 +0.023 |

86 22 +15.294 +7.211 +0.084 +0.035 |

87 10 +15.778 +6.531 +0.125 +0.023 |

88 25 +14.410 +4.750 +0.074 +0.041 |

89 16 +14.492 +4.791 +0.063 +0.022 |

90 13 +14.603 +4.228 +0.145 +0.027 |

91 28 +13.905 +5.612 +0.076 +0.033 |

92 14 +14.440 +6.275 +0.131 +0.041 |

93 47 +13.523 +7.251 +0.072 +0.038 |

94 12 +14.333 +3.856 +0.101 +0.029 |

95 16 +13.708 +5.122 +0.143 +0.024 |

96 33 +13.152 +5.027 +0.065 +0.026 |

97 30 +12.968 +4.903 +0.047 +0.025 |

98 9 +13.861 +5.248 +0.086 +0.028 |

99 10 +13.489 +4.008 +0.071 +0.019 |

100 14 +12.934 +2.692 +0.032 +0.013 |

101 17 +12.566 +4.982 +0.090 +0.025 |

102 21 +12.090 +4.914 +0.029 +0.029 |

103 9 +13.111 +6.658 +0.111 +0.030 |

104 14 +12.495 +3.870 +0.111 +0.032 |

105 48 +11.772 +6.687 +0.028 +0.027 |

106 37 +11.746 +5.791 +0.021 +0.019 |

107 59 +11.140 +6.192 +0.037 +0.025 |

108 43 +11.132 +5.181 +0.048 +0.022 |

109 12 +11.742 +3.423 +0.082 +0.016 |

110 28 +11.164 +6.144 +0.058 +0.025 |

111 17 +11.037 +3.539 +0.095 +0.016 |

112 20 +10.684 +4.240 +0.096 +0.039 |

113 17 +10.721 +4.539 +0.050 +0.017 |

114 14 +10.934 +6.356 +0.057 +0.028 |

115 17 +10.699 +4.717 +0.070 +0.018 |

116 24 +10.438 +4.069 +0.031 +0.020 |

117 36 +10.124 +5.411 +0.049 +0.033 |

118 32 +10.071 +5.457 +0.040 +0.025 |

119 14 +10.407 +5.849 +0.065 +0.029 |

120 18 +10.242 +4.660 +0.132 +0.054 |

121 7 +11.286 +3.847 +0.104 +0.017 |

122 9 +10.861 +5.445 +0.102 +0.037 |

123 20 +10.053 +4.689 +0.076 +0.030 |

124 21 +9.610 +4.807 +0.020 +0.012 |

125 19 +9.602 +5.877 +0.135 +0.043 |

126 31 +9.249 +4.785 +0.009 +0.013 |

127 26 +9.342 +4.100 +0.084 +0.029 |

128 14 +9.549 +4.153 +0.057 +0.026 |

129 38 +9.018 +5.292 +0.041 +0.023 |

130 33 +9.085 +5.062 +0.066 +0.030 |

131 20 +9.163 +3.730 +0.065 +0.025 |

132 48 +8.770 +5.326 +0.039 +0.031 |

133 43 +8.660 +4.210 +0.001 +0.001 |

134 12 +9.152 +5.241 +0.082 +0.033 |

135 41 +8.449 +4.450 +0.003 +0.005 |

136 34 +8.497 +4.159 +0.025 +0.017 |

137 25 +8.560 +4.928 +0.116 +0.039 |

138 16 +8.775 +5.055 +0.102 +0.041 |

139 12 +8.939 +2.347 +0.068 +0.029 |

140 19 +8.573 +4.811 +0.043 +0.031 |

141 24 +8.217 +4.210 +0.067 +0.042 |

142 52 +7.736 +4.654 +0.012 +0.012 |

143 31 +7.860 +4.832 +0.028 +0.021 |

144 56 +7.608 +4.576 +0.036 +0.019 |

145 13 +8.115 +3.815 +0.104 +0.029 |

146 14 +7.681 +2.466 +0.079 +0.033 |

147 30 +7.306 +3.814 +0.015 +0.011 |

148 49 +7.098 +4.127 +0.012 +0.011 |

149 33 +7.134 +3.199 +0.082 +0.040 |

150 38 +7.149 +3.712 +0.028 +0.025 |

151 6 +8.133 +4.246 +0.131 +0.030 |

152 9 +7.556 +2.518 +0.088 +0.030 |

153 61 +6.752 +4.470 +0.014 +0.014 |

154 16 +7.183 +2.766 +0.033 +0.016 |

155 45 +6.676 +4.142 +0.024 +0.017 |

156 14 +7.066 +3.451 +0.039 +0.020 |

157 24 +6.674 +3.401 +0.077 +0.020 |

158 31 +6.551 +3.936 +0.024 +0.018 |

159 27 +6.433 +3.028 +0.034 +0.030 |

160 44 +6.234 +3.101 +0.007 +0.010 |

161 47 +6.212 +3.096 +0.014 +0.016 |

162 14 +6.725 +3.043 +0.033 +0.021 |

163 35 +6.155 +3.638 +0.018 +0.012 |

164 13 +6.564 +3.278 +0.077 +0.051 |

165 26 +6.025 +3.595 +0.026 +0.019 |

166 28 +5.881 +3.529 +0.041 +0.026 |

167 14 +6.066 +2.873 +0.050 +0.027 |

168 15 +5.952 +3.521 +0.077 +0.028 |

169 13 +5.885 +2.681 +0.078 +0.025 |

170 20 +5.721 +2.070 +0.016 +0.015 |

171 14 +5.945 +2.420 +0.003 +0.004 |

172 41 +5.538 +3.236 +0.046 +0.030 |

173 19 +5.591 +3.601 +0.041 +0.026 |

174 71 +5.288 +3.162 +0.025 +0.021 |

175 75 +5.236 +3.971 +0.029 +0.018 |

176 34 +5.328 +3.509 +0.056 +0.030 |

177 10 +5.711 +2.762 +0.013 +0.006 |

178 28 +5.299 +2.910 +0.024 +0.016 |

179 37 +5.180 +2.696 +0.016 +0.013 |

180 11 +5.309 +2.258 +0.075 +0.032 |

181 10 +5.422 +2.888 +0.074 +0.028 |

182 33 +4.924 +2.649 +0.022 +0.014 |

183 10 +5.444 +2.660 +0.067 +0.034 |

184 8 +5.464 +1.106 +0.081 +0.017 |

185 16 +5.350 +3.286 +0.042 +0.027 |

186 47 +4.845 +3.440 +0.017 +0.012 |

187 10 +5.178 +2.555 +0.063 +0.016 |

188 68 +4.763 +2.642 +0.023 +0.016 |

189 32 +4.740 +2.304 +0.023 +0.016 |

190 21 +4.800 +2.779 +0.033 +0.020 |

191 46 +4.551 +3.214 +0.025 +0.019 |

192 36 +4.465 +2.389 +0.021 +0.016 |

193 47 +4.425 +2.555 +0.015 +0.012 |

194 109 +4.191 +2.477 +0.032 +0.021 |

195 16 +4.617 +2.284 +0.020 +0.013 |

196 18 +4.399 +2.586 +0.059 +0.042 |

197 35 +4.244 +2.185 +0.024 +0.013 |

198 39 +4.128 +2.393 +0.040 +0.027 |

199 36 +4.081 +2.608 +0.029 +0.016 |

200 36 +4.084 +2.709 +0.018 +0.016 |

201 15 +4.343 +1.155 +0.018 +0.025 |

202 37 +3.953 +2.368 +0.042 +0.034 |

203 28 +4.069 +2.777 +0.036 +0.021 |

204 23 +4.209 +2.883 +0.025 +0.017 |

205 17 +3.971 +3.123 +0.050 +0.025 |

206 42 +3.770 +2.281 +0.019 +0.013 |

207 24 +3.750 +2.648 +0.071 +0.030 |

208 19 +3.778 +1.544 +0.019 +0.012 |

209 33 +3.716 +1.816 +0.031 +0.021 |

210 31 +3.542 +2.095 +0.020 +0.015 |

211 30 +3.713 +1.505 +0.032 +0.019 |

212 32 +3.510 +2.100 +0.017 +0.012 |

213 29 +3.394 +2.602 +0.059 +0.034 |

214 25 +3.527 +2.174 +0.036 +0.022 |

215 46 +3.294 +2.336 +0.046 +0.022 |

216 65 +3.252 +2.118 +0.022 +0.015 |

217 35 +3.333 +1.960 +0.028 +0.012 |

218 46 +3.223 +1.736 +0.023 +0.013 |

219 50 +3.158 +1.836 +0.016 +0.014 |

220 52 +3.142 +2.102 +0.028 +0.015 |

221 46 +3.149 +1.661 +0.010 +0.011 |

222 27 +3.077 +2.145 +0.077 +0.029 |

223 40 +3.063 +1.725 +0.036 +0.018 |

224 102 +2.984 +2.087 +0.027 +0.020 |

225 27 +2.986 +1.036 +0.014 +0.013 |

226 44 +2.823 +1.508 +0.016 +0.017 |

227 25 +2.797 +1.737 +0.028 +0.013 |

228 23 +2.775 +1.695 +0.034 +0.016 |

229 17 +2.559 +1.408 +0.069 +0.036 |

230 50 +2.553 +1.813 +0.040 +0.022 |

231 38 +2.509 +1.395 +0.021 +0.012 |

232 27 +2.373 +1.461 +0.025 +0.016 |

233 17 +2.441 +1.572 +0.030 +0.019 |

234 55 +2.229 +1.468 +0.037 +0.019 |

235 22 +2.286 +1.074 +0.027 +0.013 |

236 30 +2.234 +1.256 +0.031 +0.017 |

237 31 +2.120 +0.912 +0.024 +0.013 |

238 23 +2.178 +1.343 +0.053 +0.022 |

239 63 +2.081 +1.231 +0.022 +0.012 |

240 39 +2.009 +0.796 +0.043 +0.020 |

241 42 +2.098 +1.169 +0.013 +0.006 |

242 25 +2.067 +1.056 +0.046 +0.022 |

243 28 +2.074 +1.367 +0.032 +0.014 |

244 54 +1.860 +1.184 +0.026 +0.016 |

245 60 +1.784 +1.220 +0.031 +0.020 |

246 37 +1.802 +0.974 +0.023 +0.016 |

247 56 +1.558 +1.311 +0.028 +0.012 |

248 30 +1.198 +0.461 +0.032 +0.017 |

249 33 +1.053 +0.548 +0.029 +0.015 |

**Table S3.3 Scluster metrics for 250 cluster solution with edge and vertex pruning set at a threshold of 0.2 listing cluster sizes (column 2) in decreasing order of cluster homogeneity (column 3).**

Solution ---------------------------------------------------------------------

cid Size ISim ISdev ESim ESdev |

------------------------------------------------------------------------

0 7 +54.476 +1.025 +0.789 +0.019 |

1 6 +51.800 +1.290 +0.767 +0.034 |

2 7 +48.714 +0.975 +0.586 +0.025 |

3 7 +47.762 +1.472 +0.587 +0.023 |

4 9 +45.694 +2.465 +0.613 +0.077 |

5 8 +45.321 +1.646 +0.661 +0.070 |

6 7 +45.952 +0.975 +0.558 +0.024 |

7 7 +45.619 +1.731 +0.537 +0.050 |

8 9 +43.750 +0.998 +0.430 +0.015 |

9 6 +46.667 +1.025 +0.704 +0.013 |

10 4 +51.167 +0.694 +0.822 +0.022 |

11 9 +42.556 +1.818 +0.476 +0.035 |

12 7 +43.762 +1.641 +0.512 +0.035 |

13 12 +40.636 +1.447 +0.344 +0.025 |

14 6 +43.267 +1.211 +0.534 +0.027 |

15 3 +54.333 +0.289 +0.850 +0.004 |

16 13 +38.218 +1.683 +0.332 +0.047 |

17 13 +37.949 +2.078 +0.347 +0.047 |

18 4 +45.500 +2.728 +0.586 +0.030 |

19 4 +44.667 +0.720 +0.583 +0.029 |

20 6 +39.800 +2.363 +0.485 +0.068 |

21 5 +41.300 +1.708 +0.533 +0.023 |

22 9 +36.833 +1.072 +0.344 +0.046 |

23 13 +35.205 +1.997 +0.277 +0.049 |

24 7 +37.619 +1.577 +0.408 +0.032 |

25 7 +37.524 +1.052 +0.476 +0.034 |

26 9 +36.056 +2.815 +0.453 +0.048 |

27 3 +48.000 +1.000 +0.596 +0.028 |

28 7 +37.048 +2.179 +0.361 +0.040 |

29 3 +47.667 +1.041 +0.594 +0.006 |

30 5 +39.200 +0.326 +0.442 +0.018 |

31 9 +34.889 +1.384 +0.379 +0.069 |

32 3 +46.667 +0.577 +0.565 +0.018 |

33 12 +33.091 +2.849 +0.284 +0.062 |

34 3 +45.333 +1.041 +0.602 +0.014 |

35 3 +44.667 +1.443 +0.647 +0.036 |

36 10 +32.689 +3.997 +0.379 +0.067 |

37 5 +36.800 +1.525 +0.390 +0.023 |

38 6 +35.133 +1.728 +0.456 +0.059 |

39 3 +44.000 +2.784 +0.578 +0.030 |

40 11 +32.018 +2.080 +0.243 +0.040 |

41 3 +43.333 +0.289 +0.496 +0.010 |

42 15 +30.619 +1.495 +0.170 +0.026 |

43 9 +32.056 +1.193 +0.285 +0.030 |

44 4 +38.167 +2.502 +0.545 +0.074 |

45 3 +42.667 +0.289 +0.469 +0.022 |

46 8 +31.857 +3.076 +0.422 +0.080 |

47 6 +33.200 +1.302 +0.320 +0.053 |

48 3 +41.667 +0.764 +0.446 +0.017 |

49 13 +29.051 +3.151 +0.215 +0.048 |

50 12 +29.212 +5.786 +0.354 +0.105 |

51 5 +33.500 +2.834 +0.379 +0.074 |

52 4 +35.667 +3.018 +0.457 +0.065 |

53 9 +29.833 +4.515 +0.389 +0.082 |

54 14 +28.473 +3.331 +0.173 +0.041 |

55 14 +28.319 +3.224 +0.221 +0.037 |

56 10 +28.911 +8.089 +0.304 +0.136 |

57 3 +39.000 +0.866 +0.496 +0.017 |

58 10 +28.311 +1.360 +0.159 +0.027 |

59 14 +27.044 +2.138 +0.111 +0.037 |

60 8 +28.750 +8.986 +0.480 +0.074 |

61 6 +30.067 +2.145 +0.375 +0.078 |

62 4 +33.167 +1.291 +0.469 +0.066 |

63 3 +37.000 +2.291 +0.508 +0.023 |

64 7 +28.524 +3.045 +0.326 +0.040 |

65 4 +32.500 +2.349 +0.301 +0.037 |

66 3 +36.000 +1.000 +0.555 +0.076 |

67 6 +28.600 +1.315 +0.272 +0.036 |

68 7 +27.381 +5.220 +0.312 +0.047 |

69 7 +26.810 +2.563 +0.250 +0.062 |

70 13 +24.872 +6.071 +0.347 +0.041 |

71 2 +46.000 +0.000 +0.640 +0.031 |

72 6 +27.267 +1.122 +0.218 +0.031 |

73 2 +46.000 +0.000 +0.752 +0.050 |

74 13 +24.218 +6.857 +0.311 +0.149 |

75 13 +23.859 +3.049 +0.222 +0.047 |

76 15 +23.333 +7.786 +0.208 +0.093 |

77 4 +29.167 +0.430 +0.305 +0.023 |

78 15 +23.076 +4.274 +0.176 +0.043 |

79 21 +22.386 +4.093 +0.078 +0.042 |

80 2 +43.000 +0.000 +0.595 +0.021 |

81 14 +22.824 +4.166 +0.152 +0.031 |

82 9 +23.889 +4.469 +0.228 +0.042 |

83 5 +26.600 +2.389 +0.313 +0.064 |

84 3 +32.000 +0.000 +0.398 +0.021 |

85 5 +25.900 +6.015 +0.340 +0.098 |

86 12 +22.212 +6.987 +0.346 +0.086 |

87 4 +27.000 +1.563 +0.247 +0.046 |

88 6 +24.200 +5.007 +0.327 +0.100 |

89 14 +21.473 +3.642 +0.179 +0.049 |

90 10 +21.467 +7.005 +0.183 +0.066 |

91 15 +20.686 +6.495 +0.183 +0.062 |

92 6 +22.867 +6.099 +0.260 +0.058 |

93 9 +21.194 +5.604 +0.155 +0.042 |

94 13 +20.487 +5.851 +0.210 +0.055 |

95 26 +19.403 +5.467 +0.111 +0.054 |

96 10 +20.778 +6.180 +0.280 +0.084 |

97 26 +19.375 +5.663 +0.123 +0.042 |

98 8 +21.286 +7.537 +0.302 +0.079 |

99 19 +19.503 +4.508 +0.135 +0.073 |

100 21 +19.671 +7.815 +0.209 +0.087 |

101 36 +19.005 +7.705 +0.157 +0.074 |

102 6 +21.867 +2.474 +0.220 +0.032 |

103 6 +21.467 +1.652 +0.172 +0.042 |

104 21 +18.605 +3.426 +0.037 +0.022 |

105 6 +20.933 +3.310 +0.151 +0.021 |

106 10 +19.289 +5.788 +0.249 +0.067 |

107 19 +18.298 +4.070 +0.087 +0.044 |

108 19 +17.556 +2.874 +0.014 +0.020 |

109 6 +20.200 +3.245 +0.235 +0.041 |

110 15 +17.981 +7.453 +0.187 +0.090 |

111 39 +16.947 +6.630 +0.058 +0.049 |

112 7 +18.857 +3.129 +0.135 +0.064 |

113 7 +18.857 +5.811 +0.246 +0.065 |

114 23 +16.692 +4.467 +0.076 +0.047 |

115 12 +17.591 +7.045 +0.153 +0.064 |

116 14 +17.396 +2.599 +0.040 +0.036 |

117 7 +18.476 +1.867 +0.135 +0.048 |

118 16 +16.908 +3.161 +0.048 +0.027 |

119 8 +18.357 +8.618 +0.144 +0.036 |

120 7 +18.238 +5.151 +0.122 +0.028 |

121 9 +17.611 +4.753 +0.175 +0.056 |

122 11 +16.927 +4.481 +0.104 +0.027 |

123 31 +15.935 +5.973 +0.071 +0.037 |

124 13 +16.628 +3.844 +0.235 +0.035 |

125 23 +16.012 +4.553 +0.024 +0.017 |

126 16 +16.350 +4.141 +0.116 +0.067 |

127 16 +16.300 +3.614 +0.038 +0.029 |

128 23 +15.941 +4.801 +0.076 +0.058 |

129 5 +19.100 +4.156 +0.282 +0.044 |

130 17 +16.162 +4.356 +0.064 +0.053 |

131 15 +16.286 +3.784 +0.156 +0.045 |

132 6 +17.933 +6.111 +0.197 +0.043 |

133 18 +15.902 +3.933 +0.027 +0.020 |

134 6 +17.733 +4.771 +0.167 +0.040 |

135 7 +17.048 +3.365 +0.138 +0.029 |

136 7 +17.048 +5.630 +0.196 +0.027 |

137 21 +15.667 +5.644 +0.093 +0.047 |

138 33 +14.877 +5.048 +0.022 +0.028 |

139 9 +16.278 +2.032 +0.089 +0.050 |

140 27 +14.803 +4.353 +0.002 +0.004 |

141 18 +15.163 +5.910 +0.135 +0.055 |

142 28 +14.680 +5.215 +0.129 +0.050 |

143 13 +15.731 +5.428 +0.114 +0.039 |

144 8 +15.821 +4.905 +0.157 +0.043 |

145 21 +14.895 +3.715 +0.039 +0.025 |

146 22 +14.710 +5.018 +0.056 +0.067 |

147 15 +14.581 +3.051 +0.016 +0.012 |

148 4 +18.167 +4.493 +0.272 +0.058 |

149 8 +15.714 +4.243 +0.133 +0.048 |

150 21 +14.343 +4.382 +0.111 +0.046 |

151 25 +14.130 +4.302 +0.042 +0.030 |

152 9 +15.083 +3.086 +0.059 +0.024 |

153 15 +14.171 +6.946 +0.228 +0.056 |

154 4 +17.667 +6.966 +0.366 +0.028 |

155 7 +15.810 +5.725 +0.116 +0.032 |

156 6 +15.667 +5.499 +0.194 +0.096 |

157 7 +16.238 +3.465 +0.099 +0.034 |

158 7 +15.238 +3.700 +0.210 +0.061 |

159 10 +14.422 +5.978 +0.222 +0.050 |

160 13 +14.128 +3.315 +0.091 +0.039 |

161 17 +14.191 +3.144 +0.021 +0.022 |

162 12 +14.561 +4.086 +0.132 +0.044 |

163 32 +13.236 +5.715 +0.117 +0.053 |

164 12 +14.030 +5.870 +0.097 +0.048 |

165 8 +14.643 +4.482 +0.081 +0.041 |

166 29 +13.392 +5.498 +0.059 +0.051 |

167 11 +13.964 +6.366 +0.173 +0.059 |

168 18 +13.307 +4.548 +0.104 +0.050 |

169 7 +15.571 +4.167 +0.144 +0.022 |

170 15 +13.819 +4.192 +0.043 +0.030 |

171 25 +12.957 +3.820 +0.052 +0.034 |

172 12 +14.212 +5.275 +0.113 +0.048 |

173 11 +13.655 +2.936 +0.070 +0.056 |

174 16 +12.908 +5.279 +0.269 +0.089 |

175 6 +14.333 +3.417 +0.203 +0.022 |

176 6 +14.267 +5.376 +0.180 +0.072 |

177 18 +12.948 +4.831 +0.121 +0.051 |

178 12 +13.364 +4.354 +0.119 +0.039 |

179 13 +12.744 +4.793 +0.189 +0.034 |

180 17 +12.831 +3.619 +0.057 +0.031 |

181 9 +12.944 +3.821 +0.128 +0.035 |

182 19 +12.257 +4.982 +0.128 +0.066 |

183 10 +12.489 +4.288 +0.163 +0.067 |

184 18 +12.203 +3.893 +0.058 +0.022 |

185 12 +12.136 +4.487 +0.140 +0.051 |

186 10 +12.444 +4.063 +0.179 +0.053 |

187 24 +11.732 +3.750 +0.098 +0.084 |

188 15 +11.752 +4.525 +0.141 +0.048 |

189 16 +11.742 +5.302 +0.105 +0.038 |

190 27 +11.345 +2.830 +0.005 +0.010 |

191 9 +11.917 +6.746 +0.161 +0.074 |

192 12 +12.273 +4.449 +0.066 +0.022 |

193 14 +11.297 +3.964 +0.111 +0.069 |

194 9 +11.750 +4.892 +0.140 +0.039 |

195 26 +10.960 +4.011 +0.023 +0.017 |

196 10 +12.333 +4.504 +0.099 +0.031 |

197 11 +10.964 +5.636 +0.220 +0.068 |

198 12 +11.000 +5.255 +0.150 +0.068 |

199 7 +13.095 +4.392 +0.138 +0.052 |

200 10 +10.867 +4.323 +0.115 +0.039 |

201 19 +10.497 +2.771 +0.057 +0.035 |

202 26 +10.132 +3.468 +0.011 +0.011 |

203 21 +10.071 +5.178 +0.151 +0.050 |

204 11 +10.164 +3.710 +0.238 +0.114 |

205 13 +11.179 +3.162 +0.100 +0.027 |

206 12 +10.136 +4.801 +0.220 +0.073 |

207 17 +10.338 +2.614 +0.045 +0.033 |

208 7 +11.333 +3.298 +0.136 +0.131 |

209 18 +10.333 +3.645 +0.117 +0.057 |

210 10 +10.400 +4.065 +0.156 +0.064 |

211 9 +11.139 +2.686 +0.078 +0.027 |

212 21 +9.671 +3.501 +0.142 +0.047 |

213 8 +10.393 +3.579 +0.133 +0.038 |

214 25 +9.833 +4.536 +0.067 +0.037 |

215 14 +10.286 +4.953 +0.067 +0.031 |

216 11 +10.000 +4.385 +0.147 +0.063 |

217 19 +9.678 +4.174 +0.110 +0.049 |

218 16 +9.867 +4.220 +0.086 +0.027 |

219 5 +10.800 +6.173 +0.358 +0.123 |

220 14 +9.725 +3.907 +0.136 +0.049 |

221 13 +9.936 +3.248 +0.113 +0.036 |

222 22 +9.424 +4.027 +0.101 +0.048 |

223 31 +8.918 +3.932 +0.127 +0.047 |

224 6 +10.600 +3.017 +0.171 +0.047 |

225 8 +9.429 +2.781 +0.088 +0.030 |

226 19 +9.480 +3.442 +0.014 +0.012 |

227 16 +9.158 +2.958 +0.104 +0.031 |

228 17 +9.243 +2.789 +0.021 +0.018 |

229 18 +8.314 +4.707 +0.172 +0.064 |

230 20 +8.395 +2.818 +0.072 +0.027 |

231 15 +8.400 +3.624 +0.165 +0.055 |

232 18 +7.667 +3.865 +0.237 +0.043 |

233 12 +8.394 +2.990 +0.100 +0.026 |

234 12 +7.879 +2.839 +0.142 +0.049 |

235 17 +7.676 +3.150 +0.082 +0.040 |

236 16 +7.750 +3.057 +0.046 +0.025 |

237 8 +7.857 +2.389 +0.134 +0.061 |

238 19 +7.433 +2.804 +0.024 +0.014 |

239 17 +7.875 +3.158 +0.087 +0.035 |

240 31 +7.133 +3.781 +0.118 +0.051 |

241 17 +7.721 +2.988 +0.082 +0.038 |

242 9 +7.139 +3.371 +0.123 +0.035 |

243 11 +7.327 +2.822 +0.083 +0.041 |

244 21 +6.824 +3.443 +0.090 +0.037 |

245 26 +6.649 +3.151 +0.143 +0.037 |

246 24 +6.130 +2.937 +0.178 +0.119 |

247 10 +6.733 +2.534 +0.113 +0.060 |

248 7 +6.000 +2.194 +0.250 +0.099 |

249 24 +5.746 +1.674 +0.046 +0.022 |

250 10 +5.489 +1.747 +0.094 +0.025 |

251 12 +5.561 +2.620 +0.106 +0.029 |

------------------------------------------------------------------------
